# Bistability and bifurcations in HIV-1 infection model with non-monotone responses

**DOI:** 10.1038/s41598-025-91417-x

**Published:** 2025-03-01

**Authors:** M. Pradeesh, Prakash Mani

**Affiliations:** Department of Mathematics, School of Advanced Sciences, Vellore Institute of Technology, Vellore, 632 014 India

**Keywords:** HIV infections, Applied mathematics

## Abstract

This study proposes a mathematical model for HIV-1 infection and investigates their qualitative dynamics such as stability, bistability, and bifurcation properties. The model builds on existing HIV-1 models by incorporating the effects of antiretroviral therapy (ART) and modeling immune-cell dynamics through non-monotone functional responses, capturing may help to gain insights into immune activation behaviors. Further, this study discusses the presence of bistability and bifurcation phenomena, indicating that HIV-1 infection dynamics can switch between multiple equilibriums depending on model parameters and initial conditions. To ensure the disease spread in the community, this study determines the formula to calculate the basic reproduction number for the model. Theoretically, this study performs the disease-free, immune-free, and infection steady-state analysis to determine the threshold conditions focusing on saddle-node, trans-critical and Hopf-type bifurcation relies on significant parameters. The study also works on a data-driven modeling approach to determine the appropriate population parameters of the model with the help of clinical trials performed on human patients for 15 weeks.

## Introduction

Infectious diseases have high rates of death, morbidity and severe consequences in the past and current century and evident is SARS COVID-19^1–6^. Advancement in the medical field helps to reduce the deaths and control the diseases through vaccination and antibodies^7^. Many diseases are eradicated from the human population due to understanding its nature of the spread and mode of transmission and its vulnerability. Though advancements in medical industry diseases like cancer, human immuno-deficiency virus (HIV) have high impact on the health consequences and, open for the biological explanation in terms of progress, in particular, the treatment options are available in single (or) combination to control the spread within the limit. Following that, the study focuses on the HIV-1 dynamics, where HIV-1 weakens the immune systems and gives the way for other diseases to infect, result in opportunistic diseases such as hepatitis^8^, influenza, Human T-lymphotropic virus-1 (HTLV-1)^9–12^, tuberculosis^13–15^ to devastate the immune system. Some of the available preventive and treatments such as awareness about safe-sex practices^16^, and non-HIV people who have high risks of getting the disease can take the pre-exposure prophylaxis (PrEP) and people who get the HIV-1 infection can take post-exposure prophylaxis (PEP). Besides, there is no complete cure for the HIV-1 infection, the therapies are available to block the further production of free-virions biologically, reverse transcriptase inhibitors (RTIs) and protease inhibitors (PIs). In this regard, the researchers are working to gain knowledge about disease transmission and immune system response before and after the treatments, in both fundamental and experimental aspects for investigation. This study approaches the disease transmission within the host through the fundamental aspects via mathematical modeling. Mathematical modeling is a lethal tool, to investigate the dynamics of disease transmission, which is less time complexity, cost-efficient, and easily approachable. The most commonly used modeling in the dynamics of transmission arises from ordinary differential equations (ODEs), fractional order differential equations^17^ and partial differential equations (PDEs). In terms of modeling in ODEs/PDEs the evolution of cells/cases for time are considered as compartments. In 19$^{th}$ century, the common model explored the dynamics of a relationship between the susceptible-infected-recovered (SIR) model with three compartments^18^. Later on, numerous modeling approaches have been done in physical, biological, medical, and engineering applications. The models are proposed appropriate ranges from single to multiple compartments based on the nature of the problem. Therefore, this study utilizes the modeling approach to investigate the dynamics of HIV-1 infection.

Through ODEs, considering target cells (CD$4^{+}$/ T-cells), infected cells, and immune cells in a quasi-steady state, the advantage of reducing the compartments can reduce the time-complexity in terms of optimization of model parameters. In addition to three-dimensional compartment models the effect of antiretroviral therapy is also involved in the model. Analysis of HIV-1 infection in-vivo along with therapy and immune-cell dynamics is crucial in the spread of the infection for better understanding^19,20^. Once, the therapy is stopped the viral population starts to grow which are in the undetectable levels is called viral rebound. Biologically, pre-treatment and post-treatment effects helps to identify the reasons (or) factors that triggers the viral reproduction once the therapy is stopped. Research directions will helps to provide some solution for long-term treatment options. The viral rebound of HIV-1 infection along with the effectors cell expansion for the different simplification such as viral population in the quasi-steady state, ignoring the latent reservoirs, absence of the death in the immune exhaustion were modeled and investigated^21^. Recently, the broadly neutralizing antibodies (bNAbs) are used to neutralize the HIV-1 strains is effective in treating the disease and their dynamics have been studied through modeling^22,23^.

Based on the discussion, mathematical modeling can help to understand the underlying mechanisms in various stages of disease and their corresponding immune dynamics both in vivo and in vitro. Although numerous models are available in the literature regarding HIV-1 infection, studies connecting data-driven modeling and parameter sensitive analysis are limited, the analysis that connects the data-driven modeling and biologically significant parameters is the motivation of this study. Data-driven modeling in HIV-1 can help to find an optimal set of parameters about clinical trials carried out with infected cases such as macaques and humans. In a traditional approach, model rate constants are considered the same for all patients irrespective of their characteristics and state of infection. However, data-driven models help to relate differential model solutions to the clinical trials, in which the optimal parameters can be entered^24^. In the data-driven approach the model should satisfy two main criteria such as “whether your model is structurally identifiable?”, if the different parameter set produces the same output for the model, then the model is structurally unidentifiable. Secondly, “whether the parameters are practically identifiable?”, “can parameters estimated from the actual data?” if yes then the model is practically identifiable. While handling the real data these are the major challenging in the modeling^25,26^.

Theoretically, there are two ways to analyze the dynamical system, one is local stability and second is global stability, the local stability is analyzing a particular equilibrium point by choosing the initial conditions within the neighborhood of the equilibrium. Without knowing the nature of the solution analyzing the model behavior is called as global stability. In HIV-1 dynamics the local stability analysis is helpful to investigate the treatment efficacy, viral persistence and immune response behavior while small change in the HIV-1 infection, from the chronic stage of HIV-1 to virus clearance rate or vice-versa. On the other hand, qualitative analysis such as stability, bistability, and bifurcations helps to identify significant parameters that are involved in evolution of cell population. Besides, bistability and bifurcations are crucial concepts in the dynamic behavior of biological systems, and HIV-1 infection is no exception. Bistability refers to the coexistence of two stable equilibriums in a system, while bifurcations represent qualitative changes in the system behavior as a parameter is varied^8^. The bistability in HIV-1 arises due to the complex behavior between viral and infected cells with the immune cell interactions. The bistability occurs due to the existence of the lower stable equilibrium and the higher stable equilibrium which is separated by an unstable equilibrium. In the lower equilibrium, the viral population or infected cells are maintained in the lower level, this shows the dominance of the immune cells and in the higher equilibrium, the dynamics is vice-versa. These bistabilities can be influenced by the initial viral population, transmission rate, the efficacy of the therapy and the immune response production and the target cell population growth rates.^27–29^

The bifurcations are classified as local and global bifurcations, the local bifurcation types are saddle-node bifurcation, Hopf-type bifurcation, trans-critical^30^, pitchfork bifurcations. And, the global bifurcations are homoclinic and heteroclinic, several theories and investigation tools are developed to determine the types of bifurcations^6,13,31^. Bifurcation is observed in the HIV models for the viral production delay, intracellular delays, immune responses, transmissions, therapy^32,33^. Due to existence of new equilibrium and disappearance of the equilibrium along with their switch in the stability nature are classified as forward and backward bifurcation for parameters and equilibrium behaviors. In this study, the different types of backward bifurcation are investigated for the immune-free equilibrium and certain forward bifurcation with the hysteresis effect is observed between the immune-free and endemic equilibrium^34,35^. Moreover, integrating the data-driven approaches with the qualitative analysis may provide a better understanding of stable regions, parametric thresholds, and the efficacy of single or combined ARTs in a realistic manner. In HIV-1 dynamics, the infection rate increases at low viral loads because of the more availability of viral particles in the host, at the higher viral concentration the infection rate decreases because of the target cells depletion. Hence, non-monotone incidence rate between the target cell and virions becomes necessary. There is a higher rate of immune response during the initial stage towards the infected cell in the host, the immune system may become exhausted and weaken. Hence, the immune response interaction with the infected cell considered as the non-monotone immune response. This study follows data-driven model approach to determine the model parameters, whereas most of the literature works consider the available data. This study proves a bistability behavior concerning the rate constant of logistic growth and immune response rate. Besides, the concept of stability, different types of bifurcations such as saddle-node, trans-critical, Hopf, forward and backward have discussed both analytical and computational aspects are some novelties of this study. The study is motivated by need to investigate the HIV-1 dynamics through mathematical models that incorporates the epidemiological and the immunological factors. Especially focusing the stability, bifurcation and bistability analysis to determine the conditions under which the infection exist and eradicated. The critical aspect to compare the model viral solution with the clinical viral load (VL) data after the initiation of the therapy, which helps us to identify the success rate of the therapy, resistance mechanism of the virus and the intervention strategies. Moreover, the study contributes the theoretical and real data validation in HIV-1 infection through modeling by advancing the understanding of the dynamics of HIV-1 by adding this novel feature. In this paper, “preliminaries” section briefly discusses the definitions and theorems are utilized for the study to derive the analytical results such as stability, bifurcation analysis. Section “model formulation” explains the basic growth model and its advancement in the HIV-1 dynamics by considering the infection, transmission and the immune responses. Section “Positivity and boundedness”, shows the cell populations are remains positive and boundedness for the positive initial conditions, followed by “Basic reproduction number” section derives the basic reproduction for the HIV-1 model. In “Steady state analysis” section the stability analysis for three cases of the equilibria are studied. “Bifurcation analysis” section derives the theoretical conditions for the various types of bifurcation such as saddle-node bifurcation, trans-critical bifurcation and Hopf bifurcation. The “Numerical simulations” investigates the data interpretation of the viral population, the parameter sensitive analysis for the HIV-1 model and the correlation matrix for the model parameters are interpreted biologically, followed by the existence of the immune-free equilibrium and the endemic equilibrium are shown in term of nullclines and the number of the states are tabulated for the significant parameters. The existence of the backward and forward bifurcations for the parameters observed from the sensitive analysis results are shown and discusses in details and the bistability behavior of the model for the sensitive parameters and for various initial conditions are investigated. Different contours plots are investigated concerning the solutions of the cell populations for HIV-1 model. The paper concludes by providing some biological behaviors in HIV-1 model and some future directions of this study.

## Preliminaries

This section describes the basic definitions and the preliminaries concepts which are required for stability, bistability and bifurcations

### Definition

^36^**Equilibrium points (Steady states)** Consider the differential equation is of the form

1 $$\begin{aligned} \dot{x}(t)=f(t,x(t)). \end{aligned}$$

The set of all points ${\textbf {x}}_e=\{x_1,x_2,x_3,\cdots x_e \cdots \}$ which satisfies the condition $f(t,x_e)=0$ such that $x_e \in {\textbf {x}}_e$ is called as equilibrium point (steady state).

### Definition

^37^ **Next generation matrix** The next-generation matrix approach is used to calculate the basic reproduction number for the models with at least 2 infectious compartments. Consider the heterogeneous population distinguished by age, sex, and stage of diseases, but grouped into compartments:

2 $$\begin{aligned} \dot{x}(t)=f(t,x). \end{aligned}$$

Let us consider the system *f*(*t*, *x*) can be mapped to various compartments such as target, virus, infection and immune response from the model, categorize the states into infectious and non-infectious to form the matrix as follows

3 $$\begin{aligned} \dot{x}=\left( T_{M}+T_{s}\right) x \end{aligned}$$

where the matrix $T_{M}$ corresponds to transmissions and the matrix $T_{s}$ corresponds to transitions (moving from one compartment to another) and the decay rate is also considered in the transition matrix. The epidemiological new infection is incorporated into $T_{M}$, and other events in $T_{s}$. Overall, the next-generation matrix is derived as follows

4 $$\begin{aligned} G=-T_{M}T_{s}^{-1}. \end{aligned}$$

Here, the outcome matrix *G* helps to determine the basic reproduction number $(R_0)$. The spectral radius or the dominant eigenvalue of *G* is called $R_0$.

### Definition

^38^ **Routh-Hurwitz matrix and the Hurwitz stability criterion:** Consider any real polynomial function *p*(*x*) of degree *n* as follows,

5 $$\begin{aligned} p(x)=\lambda _nx^n+\lambda _{n-1}x^{n-1}+\cdots +\lambda _{1}x+\lambda _{0}. \end{aligned}$$

To determine the stability behavior with the help of coefficients of the polynomial *p*(*x*), it can be arranged in the given tabular form

**Table 1:** Routh-Hurwitz table.

$\lambda _n$	$\lambda _{n-2}$	$\lambda _{n-4}$	$\cdots$
$\lambda _{n-1}$	$\lambda _{n-3}$	$\lambda _{n-3}$	$\cdots$
$b_1$	$b_2$	$b_3$	$\cdots$
$c_1$	$c_2$	$c_3$	$\cdots$
$\vdots$	$\vdots$	$\vdots$	$\vdots$

Notice that, Table 1 has $n+1$ rows and contains some unknown factors $b_i$ and $c_i$ ${i=1,2, \cdots ,n}$ which can be found through the following expression

$$\begin{aligned} b_{i} =\dfrac{\lambda _{n-1}\cdot \lambda _{n-2i}-\lambda _{n}\cdot \lambda _{n-(2i+1)}}{\lambda _{n-1}}\\ c_{i} =\dfrac{b_1\cdot \lambda _{n-(2i+1)}-\lambda _{n-1}\cdot b_{i+1}}{b_1}. \end{aligned}$$

Mathematically, if the entries in the first row of Table 1 are positive then eigenvalues have negative real parts, which helps to conclude that equilibrium is stable. Similarly, any sign-change in the first column of Table 1 indicates the sign change in the eigenvalues.

### Definition

^39^ **Hopf bifurcation** Consider the system of two coupled first order differential equation

6 $$\begin{aligned} \dfrac{dx}{dt}&= f(x,y,\mu ) \nonumber \\ \dfrac{dy}{dt}&= g(x,y,\mu ) \end{aligned}$$

where $\mu$ is a parameter. The system has a fixed point (equilibrium point) $(x^*, y^*)$, which may depend on $\mu$. Let the eigenvalues of the linearized system about this fixed point be given by

$$\begin{aligned} \lambda (\mu )=\alpha (\mu )+i\beta (\mu ) \text { and } \bar{\lambda }(\mu )=\alpha (\mu )+i\beta (\mu ). \end{aligned}$$

A Hopf bifurcation is a local bifurcation, where a system looses it stability and a small amplitude of oscillation occurs, when a conjugated complex pair of eigenvalues arises around the equilibrium point as crosses the boundary of stability, that is, the imaginary axis of the complex plane. Mathematically, it means that a Hopf bifurcation of the fixed point of the two dimensional system occurs at some critical value of the parameter, $\mu =\mu _c$, if the following conditions are satisfied

- $$f(x^*,y^*,\mu _c)=0$$, $$g(x^*,y^*,\mu _c)=0.$$
- The Jacobian matrix $$\begin{pmatrix} \dfrac{\partial f}{\partial x}&\dfrac{\partial f}{\partial y} \dfrac{\partial g}{\partial x}&\dfrac{\partial g}{\partial y} \end{pmatrix}_{(x^*,y^*)}$$ has a pair of purely imaginary eigenvalues $\pm i\omega$ at $(x^*,y^*,\mu _c)$, that is $(\alpha (\mu _c)=0, \beta (\mu _c)\ne 0)$.
- $$\dfrac{d\alpha (\mu )}{d\mu }\ne 0$$ at $$\mu =\mu _c.$$

** Identifying types of bifurcation**^39^ To know the qualitative behavior of the solution of the given *n*-dimensional dynamical system,

7 $$\begin{aligned} \dot{x}=\dfrac{dx}{dt}=f(x,\mu ), x\in \mathbb {R}^n \text { and } \mu \in \mathbb {R}^n. \end{aligned}$$

near the non-hyperbolic equilibrium points, changes as the vector field *f* passes through a point in the bifurcation set or as the parameter, $\mu$ varies through a bifurcation value $\mu _0$.

### Theorem

^39^**(Sotomayor’s theorem)**.

Suppose that $f(x_0,\mu _0)=0$ and the $n\times n$ matrix $A=Df(x_0,\mu _0)$ has a simple eigenvalue $\lambda =0$ with eigenvector *v* and that $A^{T}$ has a eigenvector *w* corresponding to the eigenvalue $\lambda =0.$ Furthermore, suppose that *A* has $n_1$ eigenvalues with negative real parts and $(n-n_1-1)$ eigenvalues with positive real parts and that the following conditions hold.

- If $w^{T}f_{\mu }(x_0,\mu _0)\ne 0$, $w^{T}\left[ D^2f(x_0,\mu _0)(v,v)\right] \ne 0$ the system (7) experiences a saddle-node bifurcation at the equilibrium point $x_0$ as the parameter $\mu$ passes through the bifurcation value $\mu =\mu _0.$
- If $w^{T}f_{\mu }(x_0,\mu _0)\ne 0$, $w^{T}[Df_{\mu }(x_0,\mu _0)]\ne 0$ and $w^{T}\left[ D^2f(x_0,\mu _0)(v,v)\right] \ne 0$ then system (7) experiences a trans-critical bifurcation at the equilibrium point $x_0$ as the parameter $\mu$ varies through the bifurcation value $\mu =\mu _0$
- If $w^{T}f_{\mu }(x_0,\mu _0)\ne 0$, $w^{T}[Df_{\mu }(x_0,\mu _0)]\ne 0$, $w^{T}\left[ D^2f(x_0,\mu _0)(v,v)\right] \ne 0$ and $w^{T}\left[ D^3f(x_0,\mu _0)(v,v)\right] \ne 0$ then system (7) experiences a pitchfork bifurcation at the equilibrium point $x_0$ as the parameter $\mu$ varies through the bifurcation value $\mu =\mu _0.$

### Theorem

^40^ Consider the following general system of ordinary differential equations with a parameter $\mu$

8 $$\begin{aligned} \dfrac{dx}{dt}=f(x,\mu ), f:\mathbb {R}^n\times \mathbb {R}\rightarrow \mathbb {R} \text { and } f\in \mathbb {C}^2(\mathbb {R}^n\times \mathbb {R}) \end{aligned}$$

where 0 is an equilibrium point of the system (that is $f(0,\mu )=0$ for all $\mu$) and assume

- $A_1$: $A=D_xf(0,0)=\left( \dfrac{\partial f_i}{\partial x_j}(0,0)\right)$ is the linearization matrix of the system around the equilibrium zero with $\mu$ evaluated at zero. Zero is a simple eigenvalue of *A* and other eigenvalues of *A* have negative real parts.
- $A_2$: Matrix *A* has a right eigenvector *w* and a left eigenvector *v* (each corresponding to the zero eigenvalue). Let $f_k$ be the $k^{th}$ component of *f*
  9 $$\begin{aligned} \gamma _1&= \sum _{k,i,j=1}^{n}v_kw_iw_j\dfrac{\partial ^2f_k}{\partial x_i\partial x_j}(0,0)\end{aligned}$$
  10 $$\begin{aligned} \gamma _2&= \sum _{k,i,j=1}^{n}v_kw_i\dfrac{\partial ^2f_k}{\partial x_i \partial \mu }(0,0). \end{aligned}$$
  The local dynamics of the system around zero is determined by the signs of $\gamma _1$ and $\gamma _2$.

1. Let $\gamma _1>0,$ $\gamma _2>0.$ When $\mu <0$ with $|\mu |< 1$, 0 is locally asymptotically stable and there exists a positive unstable equilibrium $0<\mu < 1$, 0 is unstable and there exists a negative, locally asymptotically stable equilibrium.
2. Let $\gamma _1<0$, $\gamma _2<0$. When $\mu <0$ with $|\mu |< 1$, 0 is unstable, when $0<\mu < 1$, 0 is locally asymptotically stable equilibrium, and there exists a positive unstable equilibrium.
3. Let $\gamma _1>0,$ $\gamma _2<0.$ When $\mu <0$ with $|\mu |< 1$, 0 is unstable, and there exists a locally asymptotically stable negative equilibrium; when $0<\mu < 1,$ 0 is stable results in a positive unstable equilibrium.
4. Let $\gamma _1<0$, $\gamma _2>0.$ When $\mu$ changes from negative to positive, 0 changes its stability from stable to unstable. Correspondingly, a negative unstable equilibrium becomes positive and locally asymptotically stable.
5. When $\gamma _1 >0$ and $\gamma _2 > 0$ , the bifurcation at $\mu = 0$ is subcritical (backward bifurcation).
6. If $\gamma _1 < 0$ and $\gamma _2 > 0$, then the bifurcation at $\mu = 0$ is supercritical (forward bifurcation).

## Model formulation

This section describes the model formulation of HIV-1 infection by considering its transmission, production of target cells, and infected cells, therapy given to the infected cells, the production of the virus from the infected cells, and the immune cell production in the host, along with their natural death rates and elimination rates. The following model (11) describes the production of the target cell population considering the logistic growth $r T(t) \left( 1 - \frac{T(t)}{K}\right)$ and the natural death rate. The basic growth model can be given as^41^

11 $$\begin{aligned} \frac{dT(t)}{dt}&= r T(t) \left( 1 - \frac{T(t)}{K}\right) - d_T T(t) \end{aligned}$$

where *r* is the intrinsic growth rate, *K* be the total carrying capacity, and $d_T$ is the target cell death rate. Once the virus enters the host, it affects the target cells at an incidence rate of $\beta$. The above model can be modified by including compartments such as infected cells *I*(*t*) and free virions *V*(*t*), these infected cells mature and start producing new virions at the rate and clearance rate^42^.

12 $$\begin{aligned} \frac{dT(t)}{dt}&= r T(t) \left( 1 - \frac{T(t)}{K}\right) -d_TT(t)-f(T(t),V(t))\nonumber \\ \frac{dI(t)}{dt}&= f(T(t),V(t)) - \delta I(t) \nonumber \\ \frac{dV(t)}{dt}&= pI(t)-c_1V(t). \end{aligned}$$

The incidence rate is a measure that scales infected cells in a cell population over some time. In Eq. (12), $f(T(t),V(t))=\dfrac{k_0T(t)V(t)^{\chi _1}}{q_1+q_2V(t)^{\chi _2}}$ be the general nonlinear incidence rate^43^, precisely, bilinear incidence $k_0T(t)V(t)$, where $k_0>0$, be constant coefficients, the nonlinear incidence rate at the saturated level is given as $\dfrac{k_0T(t)V(t)}{1+a V(t)}$, and the model with the specific nonlinear incidence rate $\dfrac{k_0V^2(t)T(t)}{1+a V^2(t)}$^44^. Once the infection starts, the immune system gets alert and starts producing the immune cells. This study considers the natural killers, especially in the general nonlinear response of effector cell populations as follows

13 $$\begin{aligned} \frac{dT(t)}{dt}&= r T(t) \left( 1 - \frac{T(t)}{K}\right) -d_TT(t)-f(T(t),V(t))\nonumber \\ \frac{dI(t)}{dt}&= f(T(t),V(t)) - \delta I(t)-f(E(t),I(t)) \nonumber \\ \frac{dV(t)}{dt}&= pI(t)-c_1V(t)\nonumber \\ \frac{dE(t)}{dt}&= f(E(t),I(t))-bE(t). \end{aligned}$$

where, *f*(*E*(*t*), *I*(*t*)) be the nonlinear immune response produced within a host due to the new infection. In reality, at first CD$4^{+}$ are targeted by the virus rapidly for a certain period. The body gets alarmed and certain physiological changes happen in the body, and the viral load spread is reduced by the immune cells, at the same time, the spread of the cell population doesn’t grow exponentially after a certain stage it has to decrease due to the crowding effect or lack of the nutrients. And the immune system can’t fight for a long time, so the immune system starts to weaken. Therefore, the incidence of the viral and CD$4^{+}$ cells and the saturated immune response are given in the form of non-monotone function, such as $\xi (I(t))=\dfrac{k_0I(t)^{\chi _1}}{q_1+q_2 I(t)^{\chi _2}}$ where $\chi _1<\chi _2$^19^. For various disease like influenza, hepatitis there is partial immune within host can be given as non-monotone incidence rate which are nonlinear^45,46^. In HIV-1 infection the immune system will take control of the virus for a period, if the disease remains untreated then the virus takes over the control and causes acquired immuno-deficiency syndrome (AIDS). To treat the HIV-1 infection the ARTs are available to control the viral load. During the initiation of the therapy, there is no further growth in the viral population, so this study assumes the viral population in a quasi-steady state, that is $V=\dfrac{pI(t)}{c_1}$. The HIV-1 infection model (13) assumes a quasi-steady state as follows^27^

14 $$\begin{aligned} \frac{dT(t)}{dt}&= r T(t) \left( 1 - \frac{T(t)}{K}\right) - d_T T(t) - \frac{\beta (1 - \epsilon ) T(t) I(t)}{1 + a I(t)^2}\nonumber \\ \frac{dI(t)}{dt}&= \frac{\beta (1 - \epsilon ) T(t) I(t)}{1 + a I(t)^2} - \delta I(t) - m I(t) E(t)\nonumber \\ \frac{dE(t)}{dt}&= \frac{c I(t) E(t)}{\eta + k E(t)^2} - bE(t). \end{aligned}$$

**Fig. 1:**
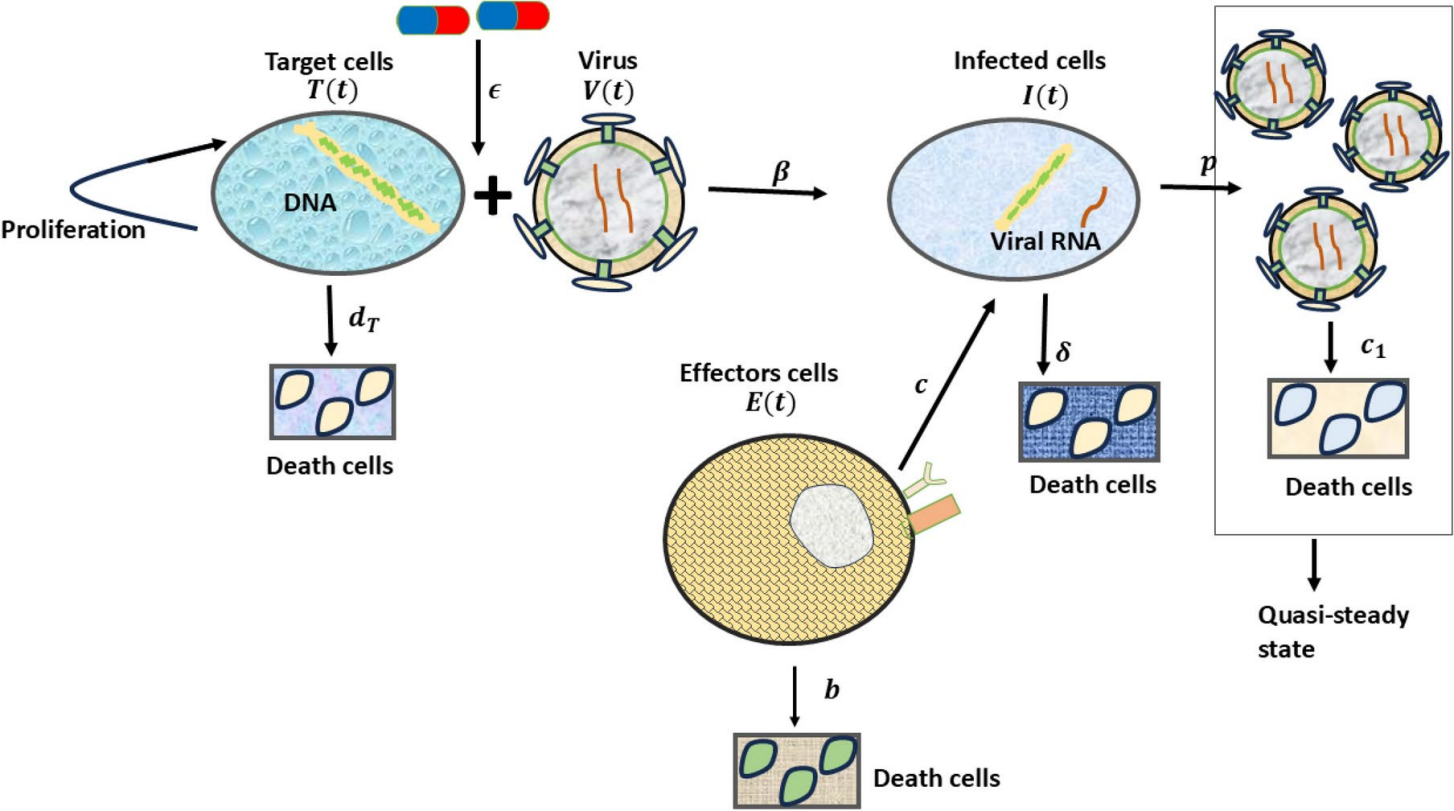
Schematic diagram for HIV-1 infection model.

Here *T*(*t*) is the target cells, *I*(*t*) is the infected cells, and *E*(*t*) is the immune response. *r* is the intrinsic growth rate, $d_T$ is the death rate, $\beta$ is the transmission rate, *K* denotes total carrying capacity, $\dfrac{I(t)}{1+aI(t)^2}$ stands for the inhibition effect due to change in the target cell population, also the crowding effect. $\epsilon$ represents anti-retroviral therapy, where the efficacy of the therapy is given by the range $0\le \epsilon \le 1$, when $\epsilon =0$ the therapy does not affect the infection, at $\epsilon =1$ the therapy works 100 $\%$ effectively, the in-between efficacy of therapy ranges are given by $0<\epsilon <1.$ $\delta$ is the death rate of the infected cell populations, *m* is the elimination rate of the infected cells by the effectors, *c* is the rate of production of the immune cell population, $\dfrac{E(t)}{\eta +kE(t)^2}$ is the inhibition of the effectors cells. *b* is the effectors cell death rate. In reality, the cell population is positive, and due to limitation such as resources, the population do not grow indefinitely. Based on this, the positivity and boundedness are derived in the following sections. Based on this, Fig. 1 represents the schematic representation of the HIV-1 infection model. The following section mathematically validates the well-possessed of *T*(*t*), *I*(*t*), and *E*(*t*) cell population through the conditions.

## Positivity and boundedness of solution

For brevity, the cell population is positive and not infinite. This section discusses the solution of the model (14) that is, the cell population remains positive and remains bounded for the positive initial conditions. The following theorem holds positivity for the HIV-1 model.

### Theorem

All the solutions of the model (14) remains non-negative for the non-negative initial conditions $T(0)>0$, $I(0)>0$ and $E(0)>0$.

### Proof

Consider the first equation of the model (14) to evaluate the non-negativity of the *T*(*t*)

15 $$\begin{aligned} \dfrac{dT(t)}{dt}&= r T(t) \left( 1 - \dfrac{T(t)}{K}\right) - d_T T(t) - \dfrac{\beta (1 - \epsilon ) T(t) I(t)}{1 + a I(t)^2} \end{aligned}$$

16 $$\begin{aligned} \dfrac{dT(t)}{dt}&\ge rT-\dfrac{rT^2}{K}-\beta (1-\epsilon ) TM-d_TT \end{aligned}$$

where $M=\max \left\{ \dfrac{I(t)}{1+aI(t)^2}\right\} =\dfrac{1}{2\sqrt{a}}.$ The incidence rate is non-monotone, hence, one can get the maximum value for the incidence and the following inequality holds,

17 $$\begin{aligned} \dfrac{dT(t)}{dt}&\ge \left\{ r-\left( d_T+\dfrac{\beta (1-\epsilon )}{2\sqrt{a}}\right) \right\} T(t)-\dfrac{rT(t)^2}{K} \nonumber \\ \dfrac{dT(t)}{dt}&\ge \dfrac{T(t)(NK-rT(t))}{K}. \end{aligned}$$

where, $N=r-\left( d_T+\dfrac{\beta (1-\epsilon )}{2\sqrt{a}}\right)$. Integrating (17), as follows

18 $$\begin{aligned} \int \dfrac{dT}{T(t)(NK-rT(t))}&\ge \dfrac{1}{K}\int dt. \end{aligned}$$

Applying the partial fraction method to simplify the Eq. (18), one can obtain

19 $$\begin{aligned} \dfrac{C_1}{T(t)}+\dfrac{C_2}{NK-rT(t)}&=\dfrac{1}{T(t)(NK-rT(t))}\end{aligned}$$

20 $$\begin{aligned} C_1(NK-rT(t))+C_2T(t)&=1. \end{aligned}$$

This leads to the equation as follows,

21 $$\begin{aligned} T(t)\ge \dfrac{BNKe^{Nt}}{1+rBe^{Nt}} = \dfrac{NBK}{e^{-Nt}+rB}\rightarrow \dfrac{NK}{r} \ge 0\hbox { as }t\rightarrow \infty . \end{aligned}$$

where $B=\dfrac{T(0)}{NK-rT(0)}.$ Thus the target cell population $T(t)>0$ $\forall$ $t>0,$ that the target cell population remains positive. Consider the immune cell population in the third equation of the model (14) as follows

22 $$\begin{aligned} \dfrac{dE(t)}{dt}&\ge cI(t)M_1-bE(t). \end{aligned}$$

Since the immune response is non-monotone, there exists a maximum value, and defining $M_1=\max \left\{ \dfrac{E(t)}{\eta +kE(t)^2}\right\} =\dfrac{1}{\sqrt{k}(\eta +1)},$ and using in Eq. (22) can lead to the following.

23 $$\begin{aligned} E(t)=E(0)e^{-bt}+M_1\int _{0}^{t}I(s)e^{b(s-t)}ds \ge 0. \end{aligned}$$

Hence, it can be seen that, $E(t)\ge 0.$ Similarly, from the second equation of the model (14), one can simplify

24 $$\begin{aligned} \dfrac{dI(t)}{dt}+\delta I(t)+mI(t)E(t)\ge \beta (1-\epsilon )T(t)M \end{aligned}$$

Applying the integration to Eq. (24), the following equation can be derived

25 $$\begin{aligned} I(t)&= I(0)e^{-\psi (t)}+\dfrac{M\beta (1-\epsilon )\int _{0}^{t}T(s)e^{\psi (t)}ds}{e^{\psi (t)}} \ge 0. \end{aligned}$$

Here $\psi (t)=\int _{0}^{t}(\delta +mE(\xi ))d\xi$. Therefore, with all the above results of *T*(*t*), *I*(*t*), and *E*(*t*) it can be concluded that all the solutions are positive. $\square$

### Theorem

The solutions of the model (14) are bounded in the given positive invariant set

$$\begin{aligned} {\Omega }=\left\{ (T(t),I(t),E(t))\in \mathbb {R}^{3}_{+}: 0<T(t),I(t)\le \dfrac{rK}{4\kappa }, 0<E(t)<\dfrac{cKr}{4b\kappa \sqrt{k}(\eta +1)}\right\} . \end{aligned}$$

### Proof

Let us consider the sum of the first two equations of model (14) as follows

$$\begin{aligned} \dfrac{d(T(t)+I(t))}{dt}&= rT(t)\left( 1-\dfrac{T(t)}{K}\right) -d_TT(t)-\delta I(t)-mI(t)E(t)\\ \dfrac{d(T(t)+I(t))}{dt}&\le \dfrac{-rT(t)^2}{K}+rT(t)-d_TT(t)-\delta I(t). \end{aligned}$$

Assume that $\kappa =\min \left\{ d,\delta \right\}$, one can get

26 $$\begin{aligned} \dfrac{d(T(t)+I(t))}{dt}+\kappa (T(t)+I(t))&\le -\dfrac{r}{K}\left( T(t)^2-KT(t)\right) \nonumber \\ \dfrac{d(T(t)+I(t))}{dt}+\kappa (T(t)+I(t))&\le -\dfrac{r}{K}\left[ T(t)^2-KT(t)+\left( \dfrac{K}{2}\right) ^2-\left( \dfrac{K}{2}\right) ^2\right] \nonumber \\ \dfrac{d(T(t)+I(t))}{dt}+\kappa (T(t)+I(t))&\le \dfrac{rK}{4}. \end{aligned}$$

Integrating (26) and taking limits as $t\rightarrow \infty$, one can get

27 $$\begin{aligned} (T(t)+I(t))\rightarrow \dfrac{rK}{4\kappa } \hbox { as, } t\rightarrow \infty . \end{aligned}$$

Thus $0\le (T(t)+I(t))\le \dfrac{rK}{4\kappa },$ $\forall$ $t\ge 0,$ from the third compartment of (14), the following expression can be derived.

28 $$\begin{aligned} \frac{dE(t)}{dt}+bE(t) = \frac{c I(t) E(t)}{\eta + k E(t)^2}. \end{aligned}$$

To find *E*(*t*), integrate the above equation and apply a limit $t\rightarrow \infty$ can lead to the following.

29 $$\begin{aligned} E(t)\le \dfrac{cKr}{4b\kappa \sqrt{k}(\eta +1)}. \end{aligned}$$

Therefore, the solutions of the model (14) are bounded. $\square$

## Basic reproduction number

Basic reproduction number $(R_0)$ is a primary tool to investigate the disease through a model. Effective measures such as awareness about the disease, spread, and control measures such as medication and quarantine are handled. The number of secondary infections caused by a single infected individual is called the basic reproduction number. The transmission and transition matrices are given as $$T_{M}=\begin{pmatrix} \dfrac{\beta (1-\epsilon )TI}{1+aI^2} \end{pmatrix}$$, and $$T_{S}=\begin{pmatrix} -\delta -mEI \end{pmatrix}$$, the transmission (new infection) and the transition matrices at equilibrium $\mathcal {E}_0$ are respectively

$$\begin{aligned} T_{M}=\begin{pmatrix} \dfrac{\beta (1-\epsilon )K(r-d_T)}{r} \end{pmatrix}, \text { and } (T_S)^{-1}=\begin{pmatrix}\dfrac{1}{\delta }\end{pmatrix}. \end{aligned}$$

From the definition of the next generation matrix, the following expression can be obtained.

30 $$\begin{aligned} T_M(T_S)^{-1}&=\begin{pmatrix} \dfrac{\beta (1-\epsilon )K(r-d_T)}{r\delta } \end{pmatrix}. \end{aligned}$$

Here, the model (14) has only one infectious compartment, that is, *I*(*t*). The $T_M$ reveals that the rate at which the number of infection cells is produced in the compartment *I*(*t*), and $T_S$ is the expected number of cell loses in the compartment *I*(*t*). Moreover, $T_MT_{S}^{-1}$ represents the expected number of secondary infections in the compartment *I*(*t*) produced by infected cells. Based on these, the basic reproduction number can be derived as in th following.

31 $$\begin{aligned} R_0= \dfrac{\beta (1-\epsilon )K(r-d_T)}{r\delta }. \end{aligned}$$

If $R_0<1$ then the disease will eventually die out in the population and if $R_0>1$ then the disease spread will possibly become a pandemic.

## Steady state analysis

This section briefly discusses the necessary and sufficient conditions that help to evaluate the dynamics of the HIV-1 infection model. Here, the model is analyzed in three different conditions, during the absence of disease, the presence of the infection but the absence of immune response, and the presence of the infection and immune responses namely, *i*) disease-free equilibrium, *ii*) immune-free equilibrium, *iii*) endemic equilibrium.

### Disease-free equilibrium

Theoretically, a disease-free equilibrium represents there is no infected cell in the population or the disease die out completely. The disease-free equilibrium $\mathcal {E}_{0}$ is given as $\mathcal {E}_{0}=(T_{0}^*,0,0)$, where, $T_{0}^{*}=\dfrac{K}{r}\left( r-d_T\right) ,$ only if $r>d_T$.

#### Theorem

The disease-free equilibrium is stable for $R_0<1$ and unstable for $R_0>1$ only if $r>d_T$.

#### Proof

The characteristic matrix evaluated at the disease-free equilibrium is given as

32 $$\begin{aligned} |J(\mathcal {E}_0)-\lambda I_{3}|=\begin{vmatrix} -(r-d_T)-\lambda&-\beta (1-\epsilon )\dfrac{K}{r}(r-d_T)&0 \\ 0&\delta (R_0-1)-\lambda&0 \\ 0&0&-b-\lambda \end{vmatrix}. \end{aligned}$$

From the above characteristic matrix (32), the eigenvalues are $\lambda _1=-(r-d_T),$ $\lambda _2=\delta (R_0-1),$ and $\lambda _3=-b,$ one can observe that the equilibrium is stable $R_0<1$, otherwise unstable. $\square$

### Immune-free equilibrium

Biologically, once the foreign agent enters the host, it starts to infect the body, the foreign agent is handled by the primary immune cells, once the risk of infection becomes high, the primary immune cells alerts the body to produce the natural killers which kills the foreign agents directly, this condition of the primary defense for the disease progression is classified as the immune-free equilibrium. In the model (14) when the cells get infected but the immune system is not yet produced, this condition is called as immune-free equilibrium $\mathcal {E}_{1}$ is given by $\mathcal {E}_{1}=(T_{1}^{*},I_{1}^{*},0)$. Since the immune-free equilibrium is challenging to find analytically, the nullcline approach is utilized, the intersection of these nullclines are the equilibrium points. The nullclines for the immune-free equilibrium from the infected compartment are given as follows

33 $$\begin{aligned} T_1^*=\dfrac{\delta (1+a (I^{*}_1)^2))}{\beta (1-\epsilon )}. \end{aligned}$$

Further, the nullcline derived from the target cell compartment of model (14) for the immune-free equilibrium can be obtained as follows

34 $$\begin{aligned} T_1^*= \dfrac{K}{r}\left( r-d_T-\dfrac{\beta (1-\epsilon )I_1^*}{1+a(I^{*}_1)^2}\right) . \end{aligned}$$

Solving these, one can get the following polynomial expression

35 $$\begin{aligned} \mathcal {A}_{1}I(t)^{4}+\mathcal {A}_{2}I(t)^{2}+\mathcal {A}_{3}I(t)+\mathcal {A}_4=0 \end{aligned}$$

where, $\mathcal {A}_{1}=r\delta a^2$ , $\mathcal {A}_2=2ar\delta -rK\beta (1-\epsilon )a+d_TK\beta (1-\epsilon )a$, $\mathcal {A}_{3}=\beta ^2(1-\epsilon )^2K$, $\mathcal {A}_{4}=r\delta -rK\beta (1-\epsilon )+d_TK\beta (1-\epsilon ).$ The derived equilibrium in (35) can be modified as in the following

36 $$\begin{aligned} a^2I(t)^4+a(2-R_0)I(t)^2-\dfrac{R_0}{r-d_T}\beta (1-\epsilon )I(t)+(1-R_0)=0 \end{aligned}$$

The outcomes of above expression mainly depends on $R_0$ provided $r>d_T.$

#### Theorem

The immune-free equilibrium is stable only if

- $$cI_1^*-b\eta <0.$$
- $$B_{1}^{2}-4B_{2}>0$$, $$B_{1}>0$$ and $$B_{2}>0.$$

#### Proof

The characteristic matrix for the Jacobian matrix evaluated at the immune-free equilibrium $\mathcal {E}_1$ is given as

37 $$\begin{aligned} { |J(\mathcal {E}_1)-\lambda I_{3}|=\begin{vmatrix} r-\dfrac{2rT_1^*}{K}-d_T-\dfrac{\beta (1-\epsilon )I_1^*}{1+a(I_1^{*})^2}-\lambda&\dfrac{-\beta (1-\epsilon )T_1^*[1-a(I_1^{*})^2]}{(1+a(I_1^{*})^2)^2}&0 \\ \dfrac{\beta (1-\epsilon )I_1^*}{1+a(I_1^{*})^2}&\dfrac{\beta (1-\epsilon )T_1^*[1-a(I_1^{*})^2]}{(1+a(I_1^{*})^2)^2}-\delta -\lambda&-m I_1^*\\ 0&0&\dfrac{cI_1^*}{\eta }-b -\lambda \end{vmatrix}}. \end{aligned}$$

From the above characteristic matrix (37) the eigenvalue $\lambda _1=\dfrac{cI_1^*}{\eta }-b$ is negative only if $cI_1^*-b\eta <0$ for the polynomial $\lambda ^2+B_{1}\lambda +B_{2}=0,$ and if $B_{1}^{2}-4B_{2}>0,$ $B_{1}>0$ and $B_{2}>0$ then the eigenvalues are negative. The coefficients are given as $B_1=-(a_{11}+a_{22})$, $B_2=a_{11}a_{22}-a_{12}a_{21}$, where $a_{11}=r-\dfrac{2rT_1^*}{K}-d_T-\dfrac{\beta (1-\epsilon )I_1^*}{1+a(I_1^{*})^2},$ $a_{12}=\dfrac{-\beta (1-\epsilon )T_1^*[1-a(I_1^{*})^2]}{(1+a(I_1^{*})^2)^2},$ $a_{21}= \dfrac{\beta (1-\epsilon )I_1^*}{1+a(I_1^{*})^2},$ and $a_{22}=\dfrac{\beta (1-\epsilon )T_1^*[1-a(I_1^{*})^2]}{(1+a(I_1^{*})^2)^2}-\delta$. $\square$

### Endemic equilibrium

In the presence of the infection along with the immune response the endemic equilibrium $\mathcal {E}_2=(T^*,I^*,E^*)$ is given as

$$\begin{aligned} T^*&= \dfrac{K}{r}\left( r-d_T-\dfrac{\beta (1-\epsilon )I^*}{1+a(I^{*})^2}\right) \\ I^*&=\dfrac{b(\eta +k(E^{*})^2)}{c} \\ E^*&= \dfrac{1}{m}\left( \dfrac{\beta (1-\epsilon )T^*}{1+a(I^{*})^2}-\delta \right) . \end{aligned}$$

Here, the endemic equilibrium $\mathcal {E}_2$ including $R_0$ can be obtained by solving the following expression

38 $$\begin{aligned}&m^2 \left[ cI(t) - b\eta + 4acI(t)^3 - 4ab\eta I(t)^2 + 6a^2cI(t)^5 - 6a^2b\eta I(t)^4 + 4a^3cI(t)^7 - 4a^3b\eta I(t)^6 - a^4b\eta I(t)^8 + a^4cI(t)^9\right] \nonumber \\&- bk\delta ^2 \left[ (R_0 - BI(t)R_0 - 1)^2 \right. - 2aI(t)^2(R_0 - BI(t)R_0 - 1)(R_0 - 2) + a^2I(t)^4 (R_0 - BI(t)R_0 - 1)\nonumber \\&\quad -a^2I(t)^4 (R_0 - 2)^2 + 2a^3I(t)^6 (R_0 - 2) - a^4I(t)^8 \left. \right] =0 \end{aligned}$$

where, $B = \beta (1-\epsilon )/(r-d_T)$. The HIV-1 dynamics is modeled using ODEs, since the model is nonlinear and analyzing nonlinear system is challenging, the study linearizes the model using Jacobian matrix approach at a particular equilibrium point. Let $\mathcal {E}_2=(T^*,I^*,E^*)$ be any arbitrary equilibrium point, then the characteristic matrix for the Jacobian matrix evaluated at the equilibrium point $\mathcal {E}_2$ is given as follows,

39 $$\begin{aligned} |J(\mathcal {E}_2)-\lambda I_{3}|=\begin{vmatrix} a_{11}-\lambda&\dfrac{-\beta (1-\epsilon )T^*[1-a(I^{*})^2]}{(1+a(I^{*})^2)^2}&0 \\ \dfrac{\beta (1-\epsilon )I^*}{1+a(I^{*})^2}&\dfrac{\beta (1-\epsilon )T^*[1-a(I^{*})^2]}{(1+a(I^{*})^2)^2}-\delta -mE^*-\lambda&-m I^*\\ 0&\dfrac{cE^*}{\eta +k(E^{*})^2}&\dfrac{cI^*[\eta -k (E^{*})^2]}{(\eta +k(E^{*})^2)^2}-b-\lambda \end{vmatrix} \end{aligned}$$

The characteristic polynomial of the above characteristic matrix (39) is given as

40 $$\begin{aligned} \lambda ^3+A_{1}\lambda ^2+A_{2}\lambda +A_3=0. \end{aligned}$$

Here, $A_{1}=-(a_{11}+a_{22}+a_{33}),$ $A_{2}=a_{22}a_{33}-a_{23}a_{32}+a_{11}a_{33}+a_{11}a_{22}-a_{12}a_{21},$ and $A_3=-\det (J(\mathcal {E}_2))$ where *J* is the Jacobian matrix. The entries in $A_1$, $A_2$ and $A_3$ are given as where $a_{11}= r-\dfrac{2rT^*}{K}-d_T-\dfrac{\beta (1-\epsilon )I^*}{1+aI^{2*}},$ $a_{12}=\dfrac{-\beta (1-\epsilon )T^*[1-aI^{2*}]}{(1+aI^{2*})^2},$ $a_{21}=\dfrac{\beta (1-\epsilon )I^*}{1+aI^{2*}},$ $a_{22}=\dfrac{\beta (1-\epsilon )T^*[1-aI^{2*}]}{(1+aI^{2*})^2}-\delta -mE^*,$ $a_{32}=\dfrac{cE^*}{\eta +kE^{2*}},$ and $a_{33}=\dfrac{cI^*[\eta -k E^{2*}]}{(\eta +kE^{2*})^2}-b.$

#### Theorem

The endemic equilibrium is stable only if all the eigenvalues are negative.

#### Proof

The characteristic matrix evaluated at the endemic equilibrium $\mathcal {E}_{2}$ as follows

41 $$\begin{aligned} |J(\mathcal {E}_2)-\lambda I_{3}|=\begin{vmatrix} a_{11}-\lambda&\dfrac{-\beta (1-\epsilon )T^*[1-a(I^{*})^2]}{(1+a(I^{*})^2)^2}&0 \\ \dfrac{\beta (1-\epsilon )I^*}{1+a(I^{*})^2}&\dfrac{\beta (1-\epsilon )T^*[1-a(I^{*})^2]}{(1+a(I^{*})^2)^2}-\delta -mE^*-\lambda&-m I^*\\ 0&\dfrac{cE^*}{\eta +k(E^{*})^2}&\dfrac{cI^*[\eta -k (E^{*})^2]}{(\eta +k(E^{*})^2)^2}-b-\lambda \\ \end{vmatrix}. \end{aligned}$$

The characteristic polynomial of the characteristic matrix (41) evaluated at the endemic equilibrium $\mathcal {E}_2$ is given as

42 $$\begin{aligned} \lambda ^3+A_{1}\lambda ^2+A_{2}\lambda +A_3=0. \end{aligned}$$

The stability of the endemic equilibrium can be analyzed utilizing the Routh-Hurwitz criterion if the following conditions are satisfied

$$\begin{aligned} A_1>0, A_1A_2-A_3>0. \end{aligned}$$

Here, $A_{1}=-(a_{11}+a_{22}+a_{33}),$ $A_{2}=a_{22}a_{33}-a_{23}a_{32}+a_{11}a_{33}+a_{11}a_{22}-a_{12}a_{21},$ and $A_3=-\det (J(\mathcal {E}_2))$, where *J* is the Jacobian matrix. The entries in $A_1$, $A_2$ and $A_3$ are given as $a_{11}= r-\dfrac{2rT^*}{K}-d_T-\dfrac{\beta (1-\epsilon )I^*}{1+a(I^{*})^2},$ $a_{12}=\dfrac{-\beta (1-\epsilon )T^*[1-a(I^{*})^2]}{(1+a(I^{*})^2)^2},$ $a_{21}=\dfrac{\beta (1-\epsilon )I^*}{1+a(I^{*})^2}$, $a_{22}=\dfrac{\beta (1-\epsilon )T^*[1-a(I^{*})^2]}{(1+a(I^{*})^2)^2}-\delta -mE^*,$ $a_{32}=\dfrac{cE^*}{\eta +kE^{2*}},$ and $a_{33}=\dfrac{cI^*[\eta -k (E^{*})^2]}{(\eta +k(E^{*})^2)^2}-b.$ $\square$

## Bifurcation analysis

While analyzing the model parameters, certain qualitative changes can occur in the model behaviors, these qualitative changes are classified as bifurcations. In this section, the model exhibits bifurcations such as saddle-node, trans-critical and Hopf bifurcations with respect to change in parameter. Theoretically, the conditions for their existence is derived. The oscillation in the dynamics of the cell population of the model is tough to analyzed, the following section helps to understand the parameters of the model which causes oscillation is cell population of the model. For the above discussion, the study performs the Hopf bifurcation analyses theoretically and simulation with respect to parameter are done for the parameters. For theoretical aspect the following theorem ensures the Hopf bifurcation,

### Hopf bifurcation analysis

To ensure the Hopf bifurcation of model (14) at the immune-free equilibrium $\mathcal {E}_{1}$, utilizing that saturated constant *a* as a bifurcation parameter.

#### Theorem

If the saturated constant rate *a* approaches the critical value $a_c,$ then proposed model (14) exhibits Hopf bifurcation around the immune-free equilibrium $\mathcal {E}_{1}$ if the following necessary and sufficient conditions are satisfied.

- $$\phi (a_c)=B_1(a_c)B_2(a_c)-B_3(a_c)=0.$$
- $$B_1(a_c)>0,$$ $$\xi _{0}^{*}=\dfrac{B_3(a_c)}{B_1(a_c)}>0.$$
- $$\left[ \dfrac{d\phi (a)}{da}\right] _{a=a_c}\ne 0.$$

#### Proof

Since all the coefficients of the characteristic equation can be expressed as a function of *a*, therefore one can write

43 $$\begin{aligned} \Phi ^{3}+B_{1}(a) \Phi ^{2}+B_{2}(a)\Phi +B_{3}(a)=0. \end{aligned}$$

The Eq. (43) has a pair of purely imaginary solutions $\Phi _{1,2}=\pm i\sqrt{(\xi _{0})},$ $\xi _{0}>0$ if and only if it can be written as

$$\begin{aligned} \mathcal {Q}(\Phi )=(\Phi ^2+\xi _{0})\mathcal {G}(\Phi ), \hbox {where}\quad \mathcal {G}(\Phi )=\Phi +\mathcal {L}_{1} \end{aligned}$$

where, $\mathcal {L}_{1},$ be the constant

44 $$\begin{aligned} \mathcal {Q}(\Phi )=\Phi ^3+\mathcal {L}_{1}\Phi ^2+\xi _{0}\Phi +\xi _{0}\mathcal {L}_{1}=0 \end{aligned}$$

Equating the coefficients of Eqs. (43) and (44) as follows

$$\begin{aligned} B_{1}=\mathcal {L}_{1}, B_{2}=\xi _{0}, B_{3} = \xi _{0}\mathcal {L}_{1}. \end{aligned}$$

From the above equation, one can get

45 $$\begin{aligned} B_{3}=\xi _{0}B_{1}. \end{aligned}$$

Thus, Eq. (43) can be written as

46 $$\begin{aligned} \Phi ^{3}+B_{1} \Phi ^{2}+\xi _{0}\Phi +\xi _{0}B_{1}=0. \end{aligned}$$

If $B_{1}B_{3}>0$, then from Eq. (45), one can have $\xi _{0}=\xi _{0}^{*},$ where

47 $$\begin{aligned} \xi _{0}^{*}= \dfrac{B_{3}}{B_{1}}. \end{aligned}$$

Substituting $\xi _{0}=\xi _{0}^{*}$ in Eq. (46) one can get the Eqs. (43) and (46) are identical if and only if, the following expression is valid

48 $$\begin{aligned} \phi =B_{1}B_{2}-B_{3}=0 \end{aligned}$$

Thus the necessary and sufficient conditions under the polynomial $\mathcal {G}(\Phi )=\Phi +B_{1}$ does not have zero solution for $B_{1}\ne 0.$ Also, the polynomial $\mathcal {G}(\phi )$ has the negative real part if $B_{1}>0.$ The positivity of $B_{1}$ gives the negative real part to the polynomial. To complete the discussion, it remains to verify the transversality condition to prove the existence of Hopf bifurcation. The function $\phi (a)$ can be expressed in the form of Orlando’s formula as follows^2^

49 $$\begin{aligned} \phi (a)=(\Phi _1+\Phi _2)(\Phi _1+\Phi _3)(\Phi _2+\Phi _3). \end{aligned}$$

Assume that $a=a_c$ (critical value). As $\phi (a_c)$ is a continuous function of all its solutions, there exists an open interval $X_{a_c}=(a_c-\epsilon , a_c+\epsilon )$, where $\Phi _1$ and $\Phi _2$ are complex conjugates $\forall$ $a\in X_{a_c}.$ Consider general form in this neighborhood as $\Phi _{1}(a)=\xi _{1}(a)+i\xi _{2}(a),$ $\Phi _{2}(a)=\xi _{1}(a)-i\xi _{2}(a)$ with $\xi _{1}(a_c)=0$, and $\xi _2(a_c)=\sqrt{\xi _{0}}>0,$ while $\text {Re}\{\Phi _{3}(a_c)\}\ne 0.$ Then, the equation becomes

$$\begin{aligned} \phi (a)&= (\Phi _1+\Phi _2)(\Phi _1\Phi _2+\Phi _2\Phi _3+\Phi _1\Phi _3+\Phi _{3}^{2})\\&=2\xi _1\left[ (\xi _1+i\xi _2)(\xi _1-i\xi _2)+(\xi _1-i\xi _2)\Phi _3+(\xi _1+i\xi _2)\Phi _3+\Phi _{3}^{2}\right] \\ \phi (a)&=2\xi _1\left[ \left( \Phi _3+\xi _1\right) ^3+\xi _{2}^{2}\right] , \phi (a_c)=0. \end{aligned}$$

Differentiating with respect to parameter *a* and substituting $``a=a_c'',$ one can get

50 $$\begin{aligned} \left[ \dfrac{d\phi (a)}{da}\right] _{a=a_c}=\left[ 2(\Phi _{3}^{2}+\xi _{2}^{2})\dfrac{d\xi _{1}(a)}{da}\right] _{a=a_c}. \end{aligned}$$

Since the solutions $\Phi _3$ have negative real parts at $a=a_c$, one can arrive at the following condition

51 $$\begin{aligned} \left[ \dfrac{d\xi (a)}{da}\right] _{a=a_c}\ne 0 \iff \left[ \dfrac{d\phi (a)}{da}\right] _{a=a_c}\ne 0. \end{aligned}$$

Thus, the transversality condition holds. From, the theorem the immune-free equilibrium $\mathcal {E}_1$ undergoes Hopf bifurcation for the saturated constant at $a=a_c$. $\square$

### Saddle-node bifurcation analysis

Our aim here is to demonstrate the behavior of saddle-node bifurcation in the solution trajectories of the model (14), for which one uses the Sotomayor theorem^39^.

#### Theorem

The endemic equilibrium of the model (14) undergoes the saddle-node bifurcation at $$c=c^{**}$$ where,

$$\begin{aligned} c^{**}=\dfrac{b(a_{11}a_{22}-a_{12}a_{21})}{a_{11}a_{22}a_{33}-a_{11}a_{33}a_{23}-a_{12}a_{21}a_{33}}. \end{aligned}$$

#### Proof

The Jacobian matrix *J* can be computed at the endemic equilibrium $$\mathcal {E}_2$$ has eigenvalue zero, if $$C_{3}(c^{**})=0$$. Let $$U=(u_1,u_2,u_3)$$ and $$W=(w_1,w_2,w_3)^T$$, sequentially represent the left and right eigenvectors of matrix *J* associated with the eigenvalue zero, for simplicity, to valid their existence, in this regard the Jacobian matrix at the endemic equilibrium is considered in the following

52 $$\begin{aligned} J(\mathcal {E}_2)=\begin{pmatrix} a_{11}& a_{12}& 0\\ a_{21} & a_{22} & a_{23}\\ 0 & ca_{32}& ca_{33}-b \end{pmatrix} \end{aligned}$$

53 $$\begin{aligned} a_{11}(a_{22}(ca_{33}-b)-ca_{32}a_{23})-a_{12}(a_{21}(ca_{33}-b))=0. \end{aligned}$$

From Eq. (53), the $$c^{**}$$ is derived, at the point the eigenvalue is zero

54 $$\begin{aligned} c^{**}&=\dfrac{b(a_{11}a_{22}-a_{12}a_{21})}{a_{11}a_{22}a_{33}-a_{11}a_{33}a_{23}-a_{12}a_{21}a_{33}}. \end{aligned}$$

The right eigenvector of zero eigenvalue for the Jacobian matrix (52) can be calculated as

$$\begin{aligned} {\left\{ \begin{array}{ll} & a_{11}u_1+a_{12}u_2=0\\ & a_{21}u_1+a_{22}u_2+a_{23}u_3=0\\ & ca_{32}u_2+(ca_{33}-b)u_3=0. \end{array}\right. } \end{aligned}$$

Therefore, to solve the above equation one can get the equations as follows

55 $$\begin{aligned} u_1=\dfrac{-a_{12}}{a_{11}}u_2, u_3=\dfrac{ca_{32}}{(b-ca_{33})}u_2, u_2&\left[ \dfrac{-a_{21}a_{12}}{a_{11}}+a_{22}+\dfrac{ca_{23}a_{32}}{(b-ca_{33})}\right] =0, u_2(0)=0. \end{aligned}$$

Here, one can observe that $u_2$ is considered as arbitrary. Further, this study assumes that $u_2=1.$ Similarly, the right eigenvector from Eq. (55) and the left eigenvector are derived as

56 $$\begin{aligned} U=\begin{bmatrix} u_1\\ u_2\\ u_3 \end{bmatrix}=\begin{bmatrix} \dfrac{-a_{12}}{a_{11}}\\ 1\\ \dfrac{ca_{32}}{b-ca_{33}} \end{bmatrix}, W=\begin{bmatrix} w_1\\ w_2\\ w_3 \end{bmatrix}^T=\begin{bmatrix} -\dfrac{a_{21}}{a_{11}}\\ 1\\ \dfrac{a_{23}}{b-ca_{33}} \end{bmatrix}^T. \end{aligned}$$

Consider, $F=\left( f_1,f_2,f_3\right)$ be the right hand-side of the model (14), the transversality conditions are derived as follows

57 $$\begin{aligned} W\left. \dfrac{dF}{dc}\right| _{c=c^{**}}=\left( \dfrac{a_{11}a_{23}+a_{12}a_{21}-a_{11}a_{22}}{ba_{11}}\right) \times \dfrac{E^*I^*}{\eta +k (E^*)^2}\ne 0 \end{aligned}$$

Similarly, the co-efficient is determined as follows

58 $$\begin{aligned}&D^2(F(\mathcal {E}_2);c^{**})(U,U)=\begin{bmatrix} \dfrac{-2r}{K}u_1^2-2\beta (1-\epsilon )\left( \dfrac{1-a(I^*)^2}{(1+a(I^*)^2)^2}\right) u_1u_2+\dfrac{2a\beta (1-\epsilon )T^*I^*[(1+a(I^*)^2)^2+2[1-a^2I^4]]}{(1+a(I^*)^2)^4}u_2^2 \\ 2\beta (1-\epsilon )\left( \dfrac{1-a(I^*)^2}{(1+a(I^*)^2)^2}\right) u_1u_2-2mu_2u_3-\dfrac{2a\beta (1-\epsilon )T^*I^*[(1+a(I^*)^2)^2+2[1-a^2I^4]]}{(1+a(I^*)^2)^4}u_2^2\\ c\left( \dfrac{\eta -k(E^*)^2}{(\eta +k(E^*)^2)^2}\right) u_2u_3-2kEcI^*\left[ \dfrac{(\eta +k(E^*)^2))^2-2(\eta ^2-k^2(E^*)^4)}{(\eta +k(E^*)^2)^4}\right] u_3^2 \end{bmatrix}\nonumber \\ &W^TD^2(F(\mathcal {E}_2);c^{**})(U,U)= -\dfrac{a_{21}}{a_{11}}\left[ \dfrac{-2r}{K}u_1^2-2\beta (1-\epsilon )\left( \dfrac{1-a(I^*)^2}{(1+a(I^*)^2)^2}\right) u_1u_2\right. \nonumber \\&\quad \left. +\dfrac{2a\beta (1-\epsilon )T^*I^*[(1+a(I^*)^2)^2+2[1-a^2I^4]]}{(1+a(I^*)^2)^4}u_2^2\right] \nonumber \\&\quad +2\beta (1-\epsilon )\left( \dfrac{1-a(I^*)^2}{(1+a(I^*)^2)^2}\right) u_1u_2-2mu_2u_3\nonumber \\&\quad -\dfrac{2a\beta (1-\epsilon )T^*I^*[(1+a(I^*)^2)^2+2[1-a^2I^4]]}{(1+a(I^*)^2)^4}u_2^2\nonumber \\&\quad +\dfrac{-mI^*(\eta +k(E^*)^2)^2}{b(\eta +k(E^*)^2)^2-c^{**}I^*(\eta -k(E^*)^2)}\left[ c^{**}\left( \dfrac{\eta -k(E^*)^2}{(\eta +k(E^*)^2)^2}\right) u_2u_3\right. \nonumber \\&\quad \left. -2kEc^{**}I^*\left[ \dfrac{(\eta +k(E^*)^2))^2-2(\eta ^2-k^2(E^*)^4)}{(\eta +k(E^*)^2)^4}\right] u_3^2\right] \ne 0. \end{aligned}$$

Hence, it is proved that the endemic equilibrium $\mathcal {E}_{2}$ undergoes the saddle-node bifurcation. $\square$

### Trans-critical bifurcation

To determine the existence of the trans-critical bifurcation, the study utilizes the center manifold theory^40^ to derive the necessary and sufficient conditions. Let us consider the model (14) as follows

59 $$\begin{aligned} f_1:=\frac{dx_1}{dt}&= r x_1 \left( 1 - \frac{x_1}{K}\right) - d_T x_1 - \frac{\beta (1 - \epsilon ) x_1 x_2}{1 + a x_2^2}\nonumber \\ f_2:=\frac{dx_2}{dt}&= \frac{\beta (1 - \epsilon ) x_1 x_2}{1 + a x_2^2} - \delta x_2 - m x_2 x_3\nonumber \\ f_3:=\frac{dx_3}{dt}&= \frac{c x_2 x_3}{\eta + k x_3^2} - bx_3 \end{aligned}$$

For convenience, to show the existence of trans-critical bifurcation, the study assumes the cell populations *T*(*t*), *I*(*t*) and *E*(*t*) as $x_1$, $x_2$ and $x_3$, also the equilibrium $T^*$, $I^*$, and $E^*$ as $x_1^*$, $x_2^*$ and $x_3^*$ for the model (14). Here $(T,I,E)=(x_1,x_2,x_3)$.

#### Theorem

The immune-free equilibrium of the model (14) undergoes the trans-critical bifurcation at $c=c^{*}=\dfrac{b\eta }{x_2^*}$. The direction of the trans-critical bifurcation is given by the quantities $\gamma _1$ and $\gamma _2$ as follows

$$\begin{aligned} \gamma _1&=\dfrac{\left( r-d_T-\dfrac{\beta (1-\epsilon )x_2}{1+ax_2^2}-\dfrac{2rx_1}{K}\right) mx_2}{-\delta \left( r-d_T-\dfrac{2rx_1}{K}-\dfrac{\beta (1-\epsilon )x_2}{1+ax_2^2}\right) +\dfrac{\beta (1-\epsilon )x_1(1-ax_2^2)}{(1+ax_2^2)^2}\left( r-d_T-\dfrac{2rx_1}{K}\right) }. \gamma _2&= \dfrac{x_2}{\eta }>0. \end{aligned}$$

if $\gamma _1>0$, then bifurcation is said to be backward, and if $\gamma _1<0,$ then it is said to be forward bifurcation.

#### Proof

The characteristic matrix calculated at the immune-free equilibrium $\mathcal {E}_1$ is given as

60 $$\begin{aligned} |J(\mathcal {E}_1)-\lambda I_{3}|=\begin{vmatrix} r-\dfrac{2rx_1^*}{K}-d_T-\dfrac{\beta (1-\epsilon )x_2^*}{1+ax_2^{2*}}-\lambda&\dfrac{-\beta (1-\epsilon )x_1^*[1-ax_2^{2*}]}{(1+ax_2^{2*})^2}&0 \\ \dfrac{\beta (1-\epsilon )x_2^*}{1+ax_2^{2*}}&\dfrac{\beta (1-\epsilon )x_1^*[1-ax_2^{2*}]}{(1+ax_2^{2*})^2}-\delta -\lambda&-m x_2^*\\ 0&0&\dfrac{cx_2^*}{\eta }-b -\lambda \\ \end{vmatrix}. \end{aligned}$$

From the Jacobian matrix (60), one can observe that the last row has eigenvalue zero if $\dfrac{cx_2^*}{\eta }-b=0,$ assume the value $c=c^*$ at which the eigenvalue becomes zero, $c^*=\dfrac{b\eta }{x_2^*}$. Now, evaluate the Jacobian matrix *J* at $c=c^*$ as follows

61 $$\begin{aligned} J=\begin{pmatrix} a_{11}& a_{12} & 0 \\ a_{21}& a_{22} & a_{23} \\ 0& 0 & 0 \end{pmatrix} \end{aligned}$$

where, $a_{11}= r-\dfrac{2rx_1^*}{K}-d_T-\dfrac{\beta (1-\epsilon )x_2^*}{1+ax_2^{2*}},$ $a_{12}=\dfrac{-\beta (1-\epsilon )x_1^*[1-ax_2^{2*}]}{(1+ax_2^{2*})^2},$ $a_{21}=\dfrac{\beta (1-\epsilon )x_2^*}{1+ax_2^{2*}},$ $a_{22}=\dfrac{\beta (1-\epsilon )x_1^*[1-ax_2^{2*}]}{(1+ax_2^{2*})^2}-\delta ,$ $a_{23}=-m x_2^*.$ The right and left eigenvector $\hat{V}=[v_1,v_2,v_3]$ and $\hat{W}=[w_1,w_2,w_3]^{T}$ calculated at the zero eigenvalue for the immune-free equilibrium is given as

62 $$\begin{aligned} \hat{V}= \begin{bmatrix} v_1\\ v_2\\ v_3 \end{bmatrix}=\begin{bmatrix} a_{12}a_{23}\\ -a_{11}a_{23}\\ a_{22}a_{11}-a_{12}a_{21} \end{bmatrix}, \hat{W}= \begin{bmatrix} w_1\\ w_2\\ w_3 \end{bmatrix}^T= \begin{bmatrix} 0\\ 0\\ 1 \end{bmatrix}^T. \end{aligned}$$

Now, the quantities $\gamma _1$ and $\gamma _2$,^13^ for model (14), are obtained as follows

63 $$\begin{aligned} \gamma _1=\sum _{i,j,k=1}^{3}w_kv_iv_j\dfrac{\partial ^2f_k}{\partial x_ix_j}, \quad \gamma _2=\sum _{i,j,k=1}^{3}w_kv_i\dfrac{\partial ^2f_k}{\partial x_i\partial c}. \end{aligned}$$

From (62), it is observed that $w_1$ and $w_2$ are zero, substituting the eigenvectors in (63) to the quantities $\gamma _1$ and $\gamma _2$, leads to the following

64 $$\begin{aligned} \gamma _1&= w_3\left( v_1v_1\dfrac{\partial ^2f_3}{\partial x_1 \partial x_1}+v_1v_2\dfrac{\partial ^2f_3}{\partial x_1 \partial x_2}+v_1v_3\dfrac{\partial ^2f_3}{\partial x_1 \partial x_3}+v_2v_1\dfrac{\partial ^2f_3}{\partial x_2 \partial x_1}+v_2v_2\dfrac{\partial ^2f_3}{\partial x_2 \partial x_2}\right. \nonumber \\&\left. \quad +v_2v_3\dfrac{\partial ^2f_3}{\partial x_2 \partial x_3}+v_3v_1\dfrac{\partial ^2f_3}{\partial x_3 \partial x_1}+v_3v_2\dfrac{\partial ^2f_3}{\partial x_3 \partial x_2}+v_3v_3\dfrac{\partial ^2f_3}{\partial x_3 \partial x_3}\right) . \end{aligned}$$

The partial derivatives for the model (14) are given as

$$\begin{aligned} \dfrac{\partial f_1}{\partial x_1}&= r-\dfrac{2rx_1^*}{K}-d_T-\dfrac{\beta (1-\epsilon )x_2^*}{1+a(x_2^{*})^2}, \dfrac{\partial f_1}{\partial x_2}= \dfrac{-\beta (1-\epsilon )x_1^*[1-a(x_2^{*})^2]}{(1+a(x_2^{*})^2)^2}, \dfrac{\partial f_1}{\partial x_1\partial x_1}= -\dfrac{2r}{K},\\ \dfrac{\partial f_1}{\partial x_1\partial x_2}&= -\dfrac{\beta (1-\epsilon )(1-a(x_2^{*})^2)}{(1+ax_{2}^*)^2}, \dfrac{\partial f_1}{\partial x_2\partial x_2}=\dfrac{\beta (1-\epsilon )x_1^*(2ax_2^*(1+a(x_2^*)^2)+4ax_2^*(1-a^2(x_2^*)^4))}{(1+a(x_2^*)^2)^4},\\ \dfrac{\partial f_2}{\partial x_1}&= \dfrac{\beta (1-\epsilon )x_2^*}{1+a(x_2^{*})^2}, \dfrac{\partial f_2}{\partial x_2}= \dfrac{\beta (1-\epsilon )x_1^*[1-a(x_2^{*})^2]}{(1+a(x_2^{*})^2)^2}-\delta -mx_3^*, \dfrac{\partial f_2}{\partial x_3}=-mx_2^*,\\ \dfrac{\partial ^2 f_2}{\partial x_2\partial x_2}&=\dfrac{-\beta (1-\epsilon )x_1^*(2ax_2^*(1+a(x_2^*)^2)+4ax_2^*(1-a^2(x_2^*)^4))}{(1+a(x_2^*)^2)^4}, \dfrac{\partial f_2}{\partial x_1\partial x_2}= \dfrac{\beta (1-\epsilon )(1-ax_2^{2*})}{1+a(x_{2}^*)^2},\\ \dfrac{\partial ^2f_2}{\partial x_2\partial x_3}&=-m , \dfrac{\partial f_3}{\partial x_2}= \dfrac{cx_3^*}{\eta +k(x_3^*)^2}, \dfrac{\partial f_3}{\partial x_3}=\dfrac{cx_2^*(\eta -k(x_3^*)^2)}{(\eta +k(x_3^*)^2)^2}, \dfrac{\partial ^2f_3}{\partial x_2\partial x_3}=c\left( \dfrac{\eta -k(x_3^*)^2}{(\eta +k(x_3^*)^2)^2}\right) ,\\ \dfrac{\partial ^2 f_3}{\partial x_3\partial x_3}&=cx_2^*\left( \dfrac{2kx_3^*(-3\eta +k(x_3^*)^2)}{(\eta +k(x_3^*)^2)^3}\right) . \end{aligned}$$

The remaining quantities are zero. Here

65 $$\begin{aligned} \gamma _1=\dfrac{\left( r-d_T-\dfrac{\beta (1-\epsilon )x_2}{1+ax_2^2}-\dfrac{2rx_1}{K}\right) mx_2}{-\delta \left( r-d_T-\dfrac{2rx_1}{K}-\dfrac{\beta (1-\epsilon )x_2}{1+ax_2^2}\right) +\dfrac{\beta (1-\epsilon )x_1(1-ax_2^2)}{(1+ax_2^2)^2}\left( r-d_T-\dfrac{2rx_1}{K}\right) }. \end{aligned}$$

Suppose $\gamma _1=0$, the following expression can be obtained

66 $$\begin{aligned} \left[ r-d_T-\dfrac{\beta (1-\epsilon )x_2}{1+ax_2^2}-\dfrac{2rx_1}{K}\right] =0. \end{aligned}$$

Simplify the above equation, and one can find the threshold value of $r^*$

$$\begin{aligned} r^*=d_T+\dfrac{\beta (1-\epsilon )x_2}{1+ax_2^2}+\dfrac{2rx_1}{K}. \end{aligned}$$

Now, based on the condition derived, the forward and backward bifurcation can be classified for $r<r^*$ and $r>r^*$

67 $$\begin{aligned} \gamma _2=\dfrac{-a_{11}a_{23}}{(a_{22}a_{11}-a_{12}a_{21})}\times \dfrac{x_3}{\eta +kx_3^2}+x_2\left[ \dfrac{\eta -kx_3^2}{(\eta +kx_3^2)^2}\right] . \end{aligned}$$

At the immune-free equilibrium the points such as $(x_1^*,x_2^*,0)$. Now, the $\gamma _2$ can be simplifies as

68 $$\begin{aligned} \gamma _2=\dfrac{x_2}{\eta }>0. \end{aligned}$$

Therefore, it can be concluded that if $\gamma _1>0$ and $\gamma _2>0$ then the bifurcation is backward, and if $\gamma _1<0$ then the bifurcation is forward depending on the nature of positive or negative signs^34^. $\square$

**Fig. 2:**
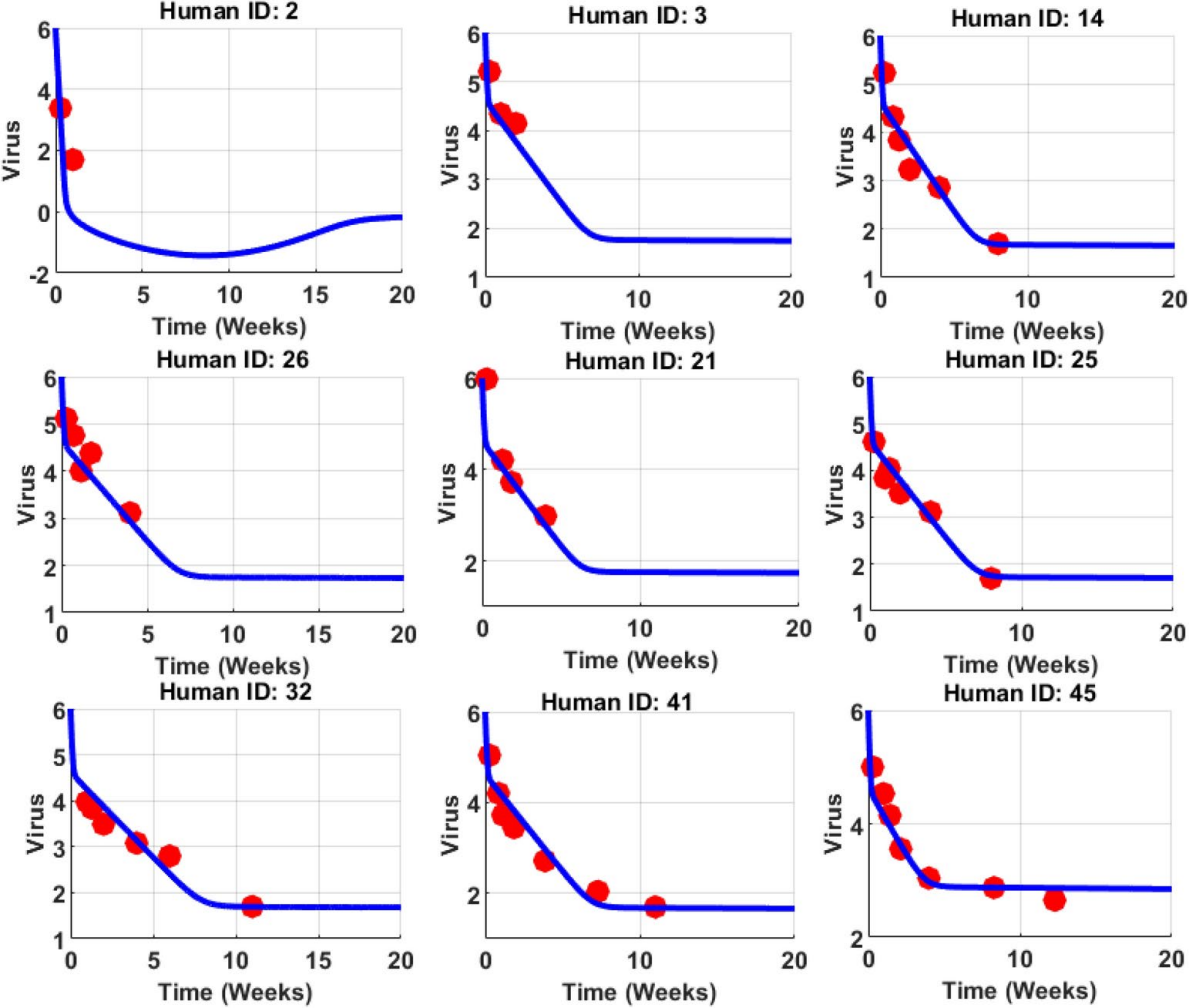
The clinical trials were performed on human patients to monitor their viral load under ART. The red dots represent the VL in natural logarithmic numbers and the blue line is the solution of the viral population. The clinical data is obtained from the literature^24^. It can be seen that VL starts declining once the therapy is initiated, and for some cases, like Human ID:2 there is an immediate response, but the response is slow in human IDs 14, 26, 21, 25, 32 41, and 45. Also, it takes more than 10 weeks for Human IDs: 32, 41, and 45. These results clearly indicate that the efficacy of ARTs are varying for each individual case and cannot be assumed as fixed.

## Numerical simulations

The objective of this section is to evaluate the rate of changes in the cell populations such as target cells, immune cells, and viral load with respect to time. In this regard, the first step is the identification of the model parameters. The parameters can be chosen from the literature works focusing on HIV-1 disease progression but the main drawback in choosing the parameters without involving the present model characteristics. To overcome this drawback, the present study follows the data-driven approach in which the model parameters can be determined through mapping the model characteristics and clinical data with the help of MONOLIX software. The following subsection briefly discusses the model identification and biological feasibility through clinical-trials.

### Data interpretation

This subsection contains the process of mapping between theoretical solutions and clinical data. Initially, the clinical data is extracted from the literature^24^, in which 48 infected individuals undergo ART for the period of 15 weeks, and their viral load was continuously monitored and recorded. The goal is to develop a differential model capable of capturing the clinically recorded viral-load dynamics, enabling the determination of ART efficacy and natural killer response over time. With the help of Monolix software, the data-fitting process has been made and the outcomes are graphically illustrated in two-dimension plot, refer Fig. 2. In detail, from Fig. 2, it can seen that, the red dots represents the clinically recorded data of viral load in individuals after therapy initiation. Biologiclaly, the viral load start declines in the patients under ARTs. The blue-line in Fig. 2 represents the solution of the model and it can be concluded that the proposed three-dimensional model can capture the dynamics of viral load in the individuals effectively. Based on these results, the suitable parameters are extracted from the software and provide in Table 2.

**Table 2:** Parameter descriptions and its ranges.

Parameter	Description	Fitting values	Biological values
*r*	Intrinsic growth rate	4.38	0.03/day^47^
*K*	Total carrying capacity	$10^5$	$10^5$cells/mL/day^47^
$d_T$	Target cell death rate	0.01	0.01/day^21^
$\beta$	Transmission rate	0.000015	$1.5\times 10^{-8}$ mL/day^47^
$\epsilon$	Drug efficacy	0.9	[0, 1]^22^
*a*	Saturated constant	0.1	0.1
*p*	New virions production	2000	2000/day^47^
$\delta$	Death rate of infected cells	1	1/day^22^
$c_{1}$	Virions clearance rate	23	23/day^47^
*m*	Infected cell elimination rate	0.00046	0.0045 mL/cells/day^47^
$\eta$	Immune impairment constant	0.0012	0.1
*k*	Immune saturated constant	0.052	–
*c*	Immune cell production	0.000071	0.1/ day^47^
*b*	Death rate	5	2/day^22^

### Parameter sensitivity analysis

This subsection demonstrates the process involved in the identification of model parameters which play a significant role in the disease progression. Partial rank correlation coefficient (PRCC) is a metric helps to analyse how strongly each model parameter correlates with the dynamics of target, infected and immune-cells dynamics. In this regard, the statistical analysis, say, PRCC metric is employed to to measures the nonlinear but monotonic relation between the model parameters through the formula

69 $$\begin{aligned} \text {PRCC}=\frac{\text {Cov}(R_x,R_y)}{\sqrt{\text {Var}(R_x)\cdot \text {Var}(R_y))}}, \end{aligned}$$

where $R_{x}, R_y$ are the rank residuals of the parmeters *x*, *y*,  respetcively. For one set of rank residuals the covariance is given as $\text {Cov}(R_x,R_y) = \mathbb {E}\left[ (R_x - \mu _{R_x})(R_y - \mu _{R_y})\right] ,$ $\mathbb {E}$ is the expectation in the probability theory. For the $n-$samples, the covariance is calculated through the formula’s

70 $$\begin{aligned} \text {Cov}(R_x,R_y)= & \frac{1}{(n-1)} \sum _{i=1}^n (R_{x_i} - \mu _{R_x})(R_{y_i} - \mu _{R_y}),\end{aligned}$$

71 $$\begin{aligned} \text {Var}(R_x)= & \frac{1}{(n-1)}\sum _{i=1}^n(R_{x_i} - \mu _{R_x})^2,\end{aligned}$$

72 $$\begin{aligned} \text {Var}(R_y)= & \frac{1}{(n-1)}\sum _{i=1}^n(R_{y_i} - \mu _{R_y)}^2. \end{aligned}$$

**Fig. 3:**
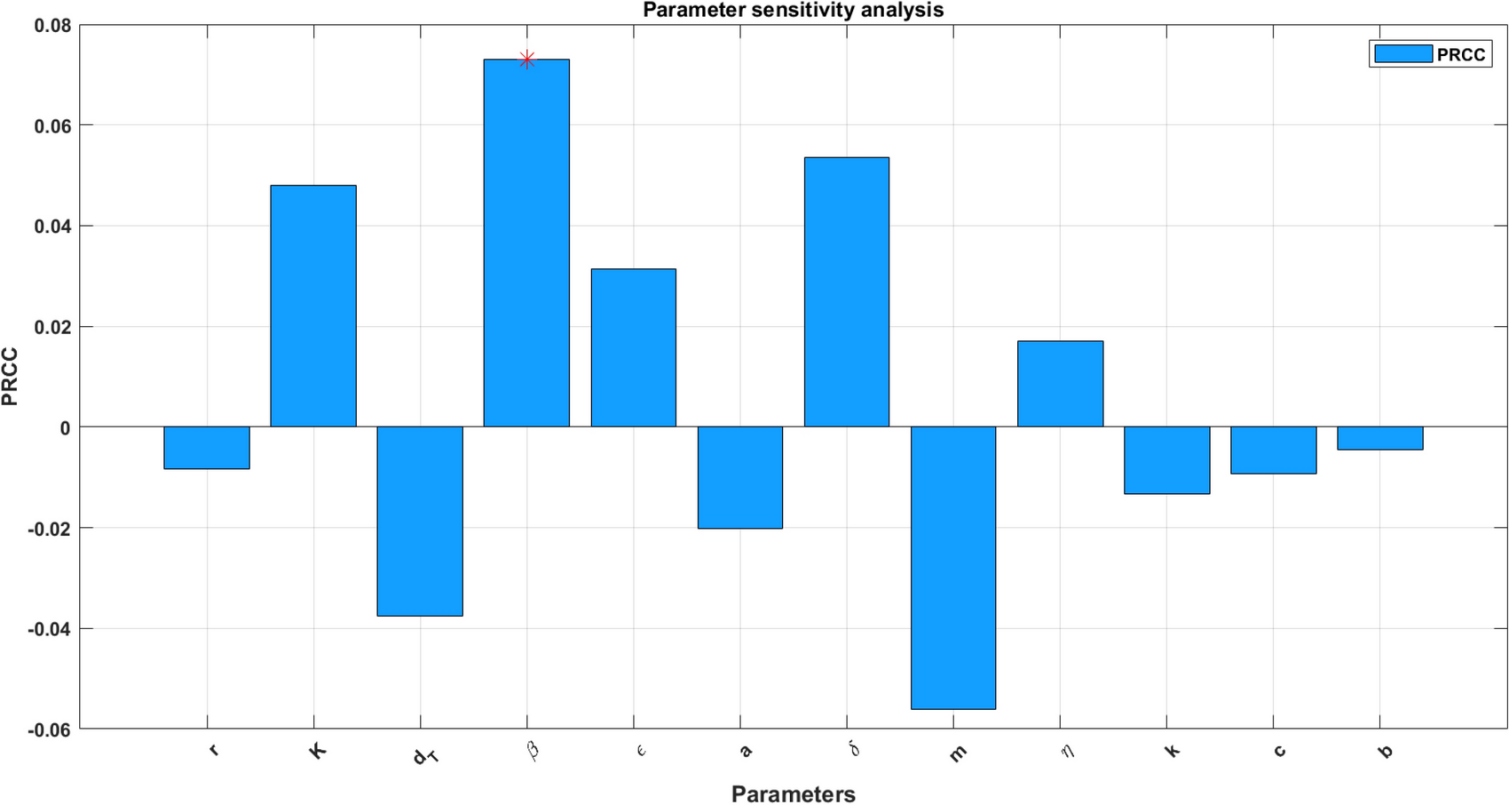
Parameter sensitivity analysis for model (14).

**Fig. 4:**
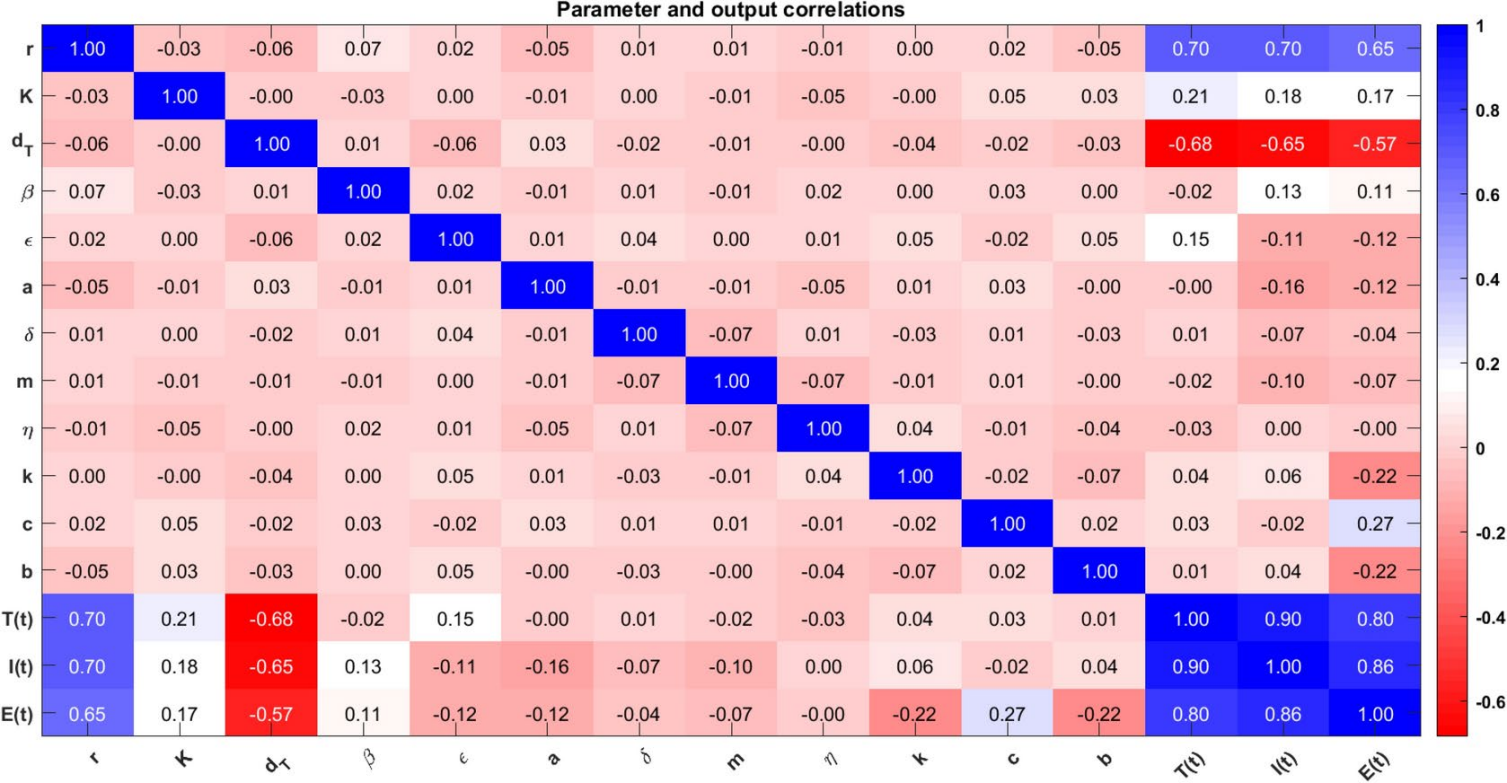
Correlation analysis and its outcomes for parameters in model (14).

Here $\mu _{R_x}, \mu _{R_y}$ are the average values of the rank residuals. The process of finding these rank residuals starts with the raw-data of model parameters $r, K, \beta , d_T, \epsilon , a, \delta , m, \eta , k, c, b$ and each coefficient stands as rate constant of *T*(*t*), *I*(*t*), *E*(*t*). The difference between the actual assigned ranks and the predicted ranks (multiple linear regression) helps to determine the rank residuals. This statistical analysis helps to measure the significance in terms of numerical value $-1$ to 1. If PRCC is 1 then for perfect positive correlation with the state variables. This study performs the PRCC measures by considering all the model parameters to identify the significance and the outcomes are depicted in Fig. 3. Biologically, infection rate $\beta$ is positively correlated with infected cells *I*(*t*), *c* is positively correlated with immune cells *E*(*t*) and $\epsilon$ is positively correlated with target cells *T*(*t*). On the otherhand, $\delta$ is negatively correlated with *I*(*t*),  the death rate *b* is negatively correlated with *E*(*t*) and $\beta$ is negatively correlated with *T*(*t*). In order to analyse the correlation between the model parameters the matrix (4) has been determined and presented in Fig. 4.

The dimension of a matrix is $15 \times 15$, which indicates the relationships between parameters and outcomes of the HIV-1 model. All the model parameters from model (14) are considered along with *T*(*t*), *I*(*t*) and *E*(*t*). The correlation coefficient is calculated through the formula

73 $$\begin{aligned} \text {PRCC}=\frac{\text {Cov}(i,j)}{\sqrt{\text {Var}(i)\cdot \text {Var}(j))}}. \end{aligned}$$

**Fig. 5:**
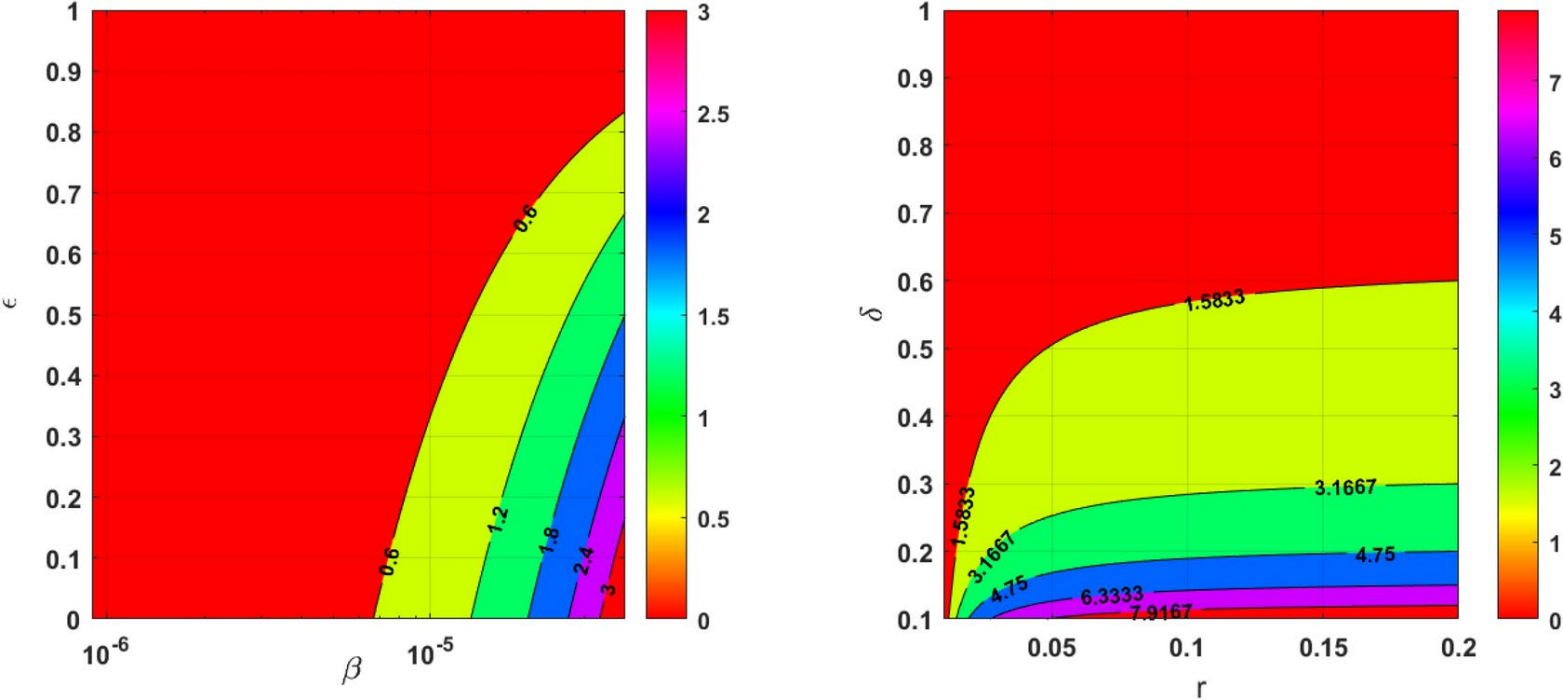
The colored regions indicate the virulence of the disease spread following the expression for $R_0$ in (31). The red color represents the $R_0<1$, where there is less risk of the disease or at the early stage of the infection, the yellow color represents the disease transmission starts to spread and the light green and blue color indicates the disease spread is moderate and precautions such as therapy are necessary to treat the disease. Further, the pink color indicates the spread of the disease lead to a chronic stage.

Here (*i*, *j*) represents the position in the matrix of Fig. 4 and each entry (*i*, *j*) explains how model parameters *i* correlate with the cell populations *j*. The colors helps to understand the correlation, say, dark colors represent a strong positive or negative correlation. For instance, one can see the strong correlation between the growth rate *r* and the target cells with a coefficient value of 0.70. The values close to 1 are strongly correlated (blue), biologically, increase in parameters will result in increased cell populations and vice-versa for negative correlation (red). Those that are negatively correlated which are close to $-1$ have an increase in parameter, decrease the other parameters, and vice-versa. The matrix shows that parameters such as *r*, $\beta$, and $\epsilon$ have a higher influence over the model behaviors. Now, from the matrix (4) one can identify the significant parameters that has the potential to bring the oscillations in cell populations and may affect the outcomes of the treatment. To an evident, the basic reproduction number $R_0$ is calculated for different ranges of the parameters $\beta , \epsilon , r, \delta$, and the outcomes are illustrated in Fig. 5. For the analysis, the remaining parameters are chosen as $K = 10^5,$ $d_T = 0.01,$ $m = 0.1,$ $k = 0.1,$ $b = 0.1,$ and $c = 0.1.$

The outcomes validate the proposed significant parameters. Mathematically, the saturation coefficient $1/(1+a I(t)^2)$ prevents irrational growth. Biologically, the model outcomes show that the high efficiency in ART may help to control the viral load.

**Table 3:** Parameter values for bifurcation analysis.

**Parameters**	*r*	*K*	*p*	$\delta$	$d_T$	$c_1$	$\beta$	$\epsilon$	*a*	*c*	*m*	$\eta$	*k*	*b*
**Values**	0.058	$10^5$	2,000	1	0.01	23	(0, 1)	[0, 1]	(0, 1)	(0, 1)	0.1	0.1	0.1	(0, 2)

### Immune-free equilibrium

**Fig. 6:**
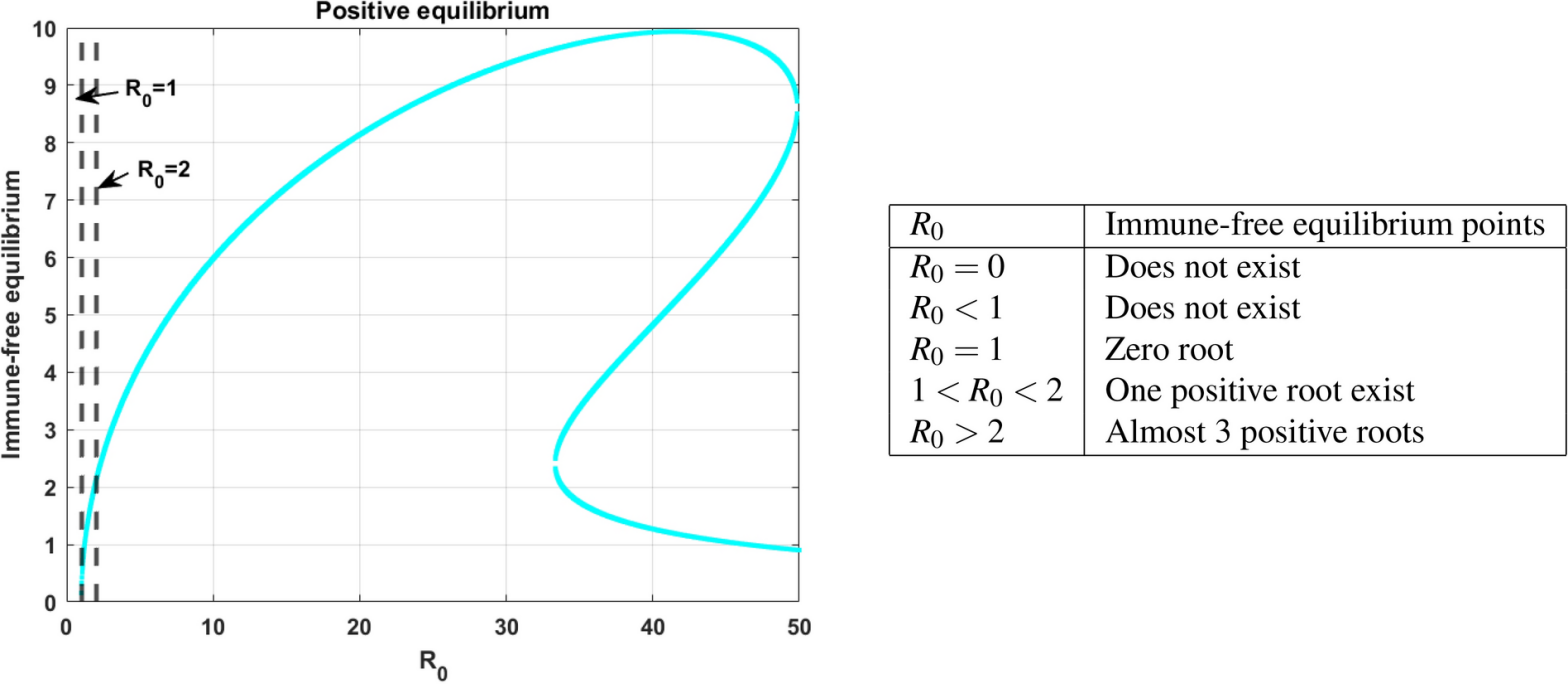
Existence of positive immune-free equilibrium with respect to $R_0$ and with an infectivity range $\beta \in (0,0.5),$ and fixed efficacy in ARTs $\epsilon = 0.99$.

This section briefly analyses the effect of immune-free equilibrium in terms of model (14). In this regard, consider the polynomial of degree four (36) with rate constants, $a, r, R_0$ and *c*. Initially, start the analysis with respect to $R_0.$ For better clarity, the effect $R_0$ is shown in Fig. 6, which clearly explains the existence of immune-free equilibriums that can be categorized into intervals, and the relationship between $R_0$ and the number of positive immune-free equilibriums are also tabulated adjacently. Biologically, the more positive equilibrium implies the higher risk of disease persisting. The number of equilibriums and their stability helps to gain insight into the overall behavior of the model, the system may exist in the multiple stable states for the parameters and the initial conditions.

**Fig. 7:**
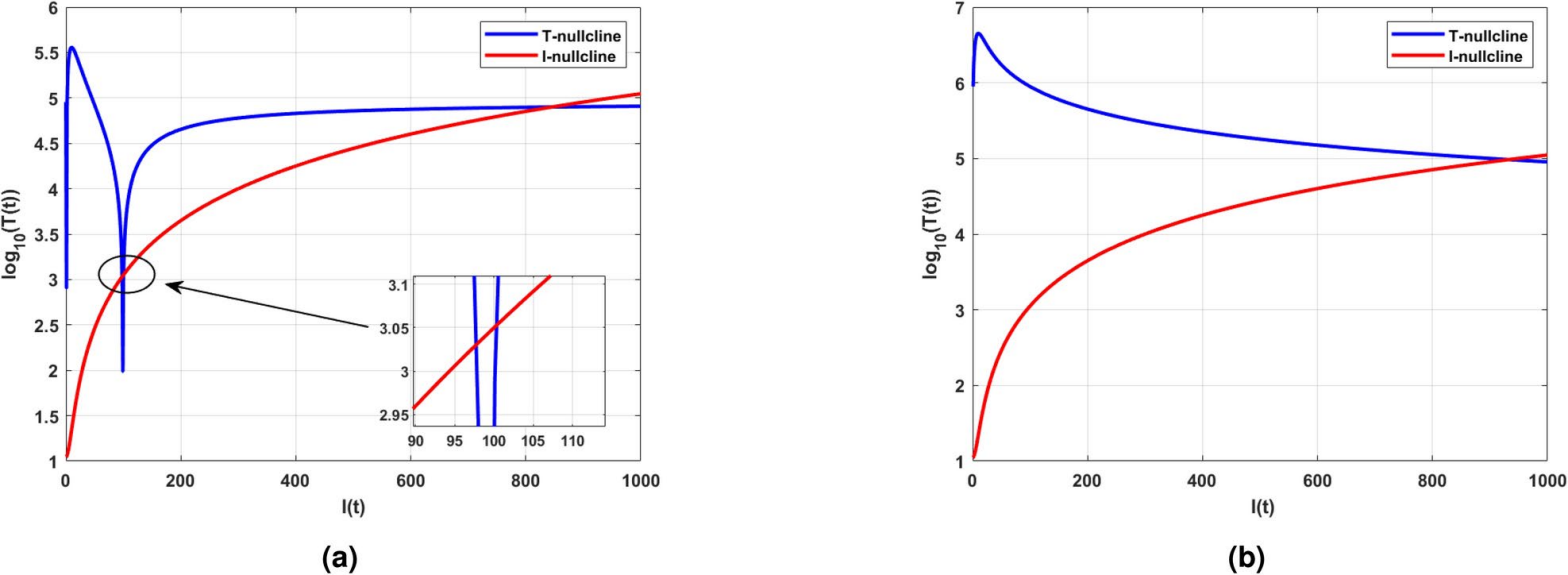
(**a**) shows the existence of three positive equilibria for the parameter $r = 0.1$. (**b**) depicts the one equilibria for $r = 0.01$. (**a**) and (**b**) are evident in the creating and destroying nature of equilibrium points for intrinsic growth rate.

Due to nonlinear interactions between the cell populations, finding the explicit expression for steady-state analysis is complex. As an alternative, nullclines are the curves that help to visualize the flow of trajectories used to analyze the nonlinear models, which are challenging and time-consuming to analyze analytically. Graphically, the intersection of the nullcline curves are called an equilibrium points. The number of equilibrium points helps to gain insights about the evolution of cell populations in various conditions or stages. Besides, the existence of equilibriums can be confirmed with the positive region of the plane in Fig. 7a and b.

**Fig. 8:**
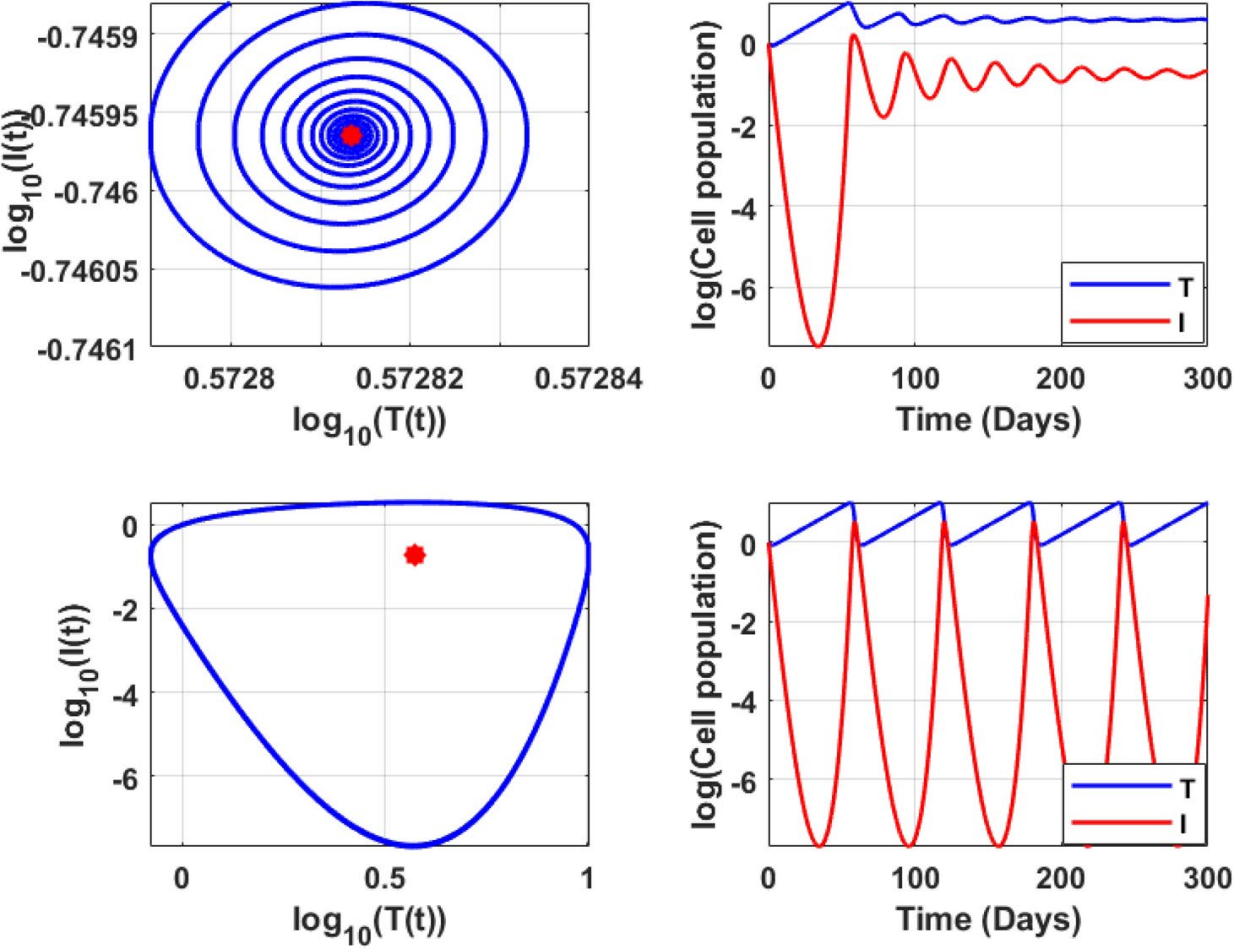
The top figures shows phase portrait and time series of lower stable equilibrium immune-free equilibrium for $a=0.1$ and bottom figures shows the phase portrait and time series of Hopf bifurcation in the immune-free equilibrium for $a=0.001$.

Mathematically, when the intrinsic growth rate $r=0.1,$ is considered and for that, it can be observed that there are three positive immune-free equilibria. On the other hand, decreases in growth rate will diminish the equilibrium to one. Likewise, a detailed analysis was performed, and the parameters are tabulated in Table 3. Followed by the eigenvalue analysis of the immune-free equilibrium with respect to the parameters *a* and *c* is given in Table 4. Also, Table 4 ensures the stability theoretical conditions for immune-free equilibrium, such as $H_1:=$ $cI_1^*-b\eta <0$, $H_2:=B_{1}^{2}-4B_{2}>0,$ $B_{1}>0$ and $B_{2}>0$. Figure 8 shows the phase portrait and the time series for the model (14).

**Table 4:** Eigenvalue analysis for immune-free equilibrium concerning $H_1:=cI_1^*-b\eta$.

Equilibrium type	Parameter	Equilibrium	Eigenvalues	Behavior
Immune-free	$a=0.001$	(3.7038; 0.1778; 0)	$(0.0778; \pm 0.2191i), H_1 > 0$	Unstable
Immune-free	$a = 0.1$	(3; 0.1; 0)	$(0.0003; \pm 0.0022i), H_1 > 0$	Unstable
		(1, 623; 76; 0)	$(0.7627; -0.0202; 0.0002), H_1 > 0$	
		(70, 432; 504; 0)	$(5.0343; -0.0200; -0.0004), H_1 > 0$	
Immune-free	$c=0.001$	(4; 0.1; 0)	(-0.0982;-0.0032 ± 0.2184i), $H_1 < 0$	Monostable
		(1, 203; 57; 0)	$(0.4690; -2.0172; 0.0226), H_1 > 0$	
		(72, 217; 442; 0)	$(4.3156; -2.0030; -0.0388), H_1 > 0$	
Immune-free	$c=0.1$	(4; 0.1; 0)	$(0.08; \pm 0.22i), H_1 > 0$	Unstable
		(1, 203; 57; 0)	$(56.80; + 0.0000i \pm 02.02), H_1 > 0$	
		(72, 217; 442; 0)	$(441.46; -2; -0.04), H_1 > 0$	

### Endemic equilibrium

**Fig. 9:**
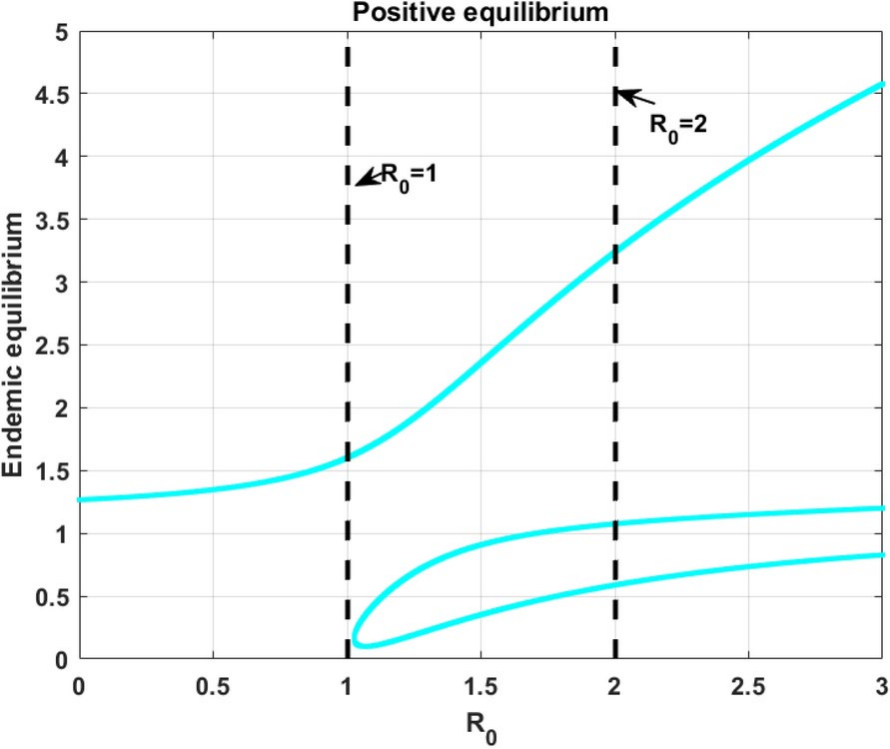
The existence of endemic equilibria with respect to the $R_0$ and the model parameters $r = 0.0141, K = 10^5, \beta = 0.007,$ $\epsilon = 0.62,$ $a = 0.1, c = 0.1$.

In this subsection, the numerical investigation of endemic equilibrium for the Eq. (38) is discussed. Based on the Eq. (38), Fig. 9 shows the positive endemic equilibrium of the model (14). It is observed that, for $R_0<1$ there exist one endemic equilibrium. For $R_0>1,$ there is an emergence of two new equilibrium, shows the complexity of the model. This is a behavior similar to the backward bifurcation. It is evident that, $R_0$ is not suffice enough to determine the disease progression. For analysis this section utilizes the parameters in the Table 3. From the sensitive analysis, the other two parameters saturated constant *a* and the immune response production rate *c*. The number of endemic equilibriums for both of these parameters are discussed in Table 5.

**Fig. 10:**
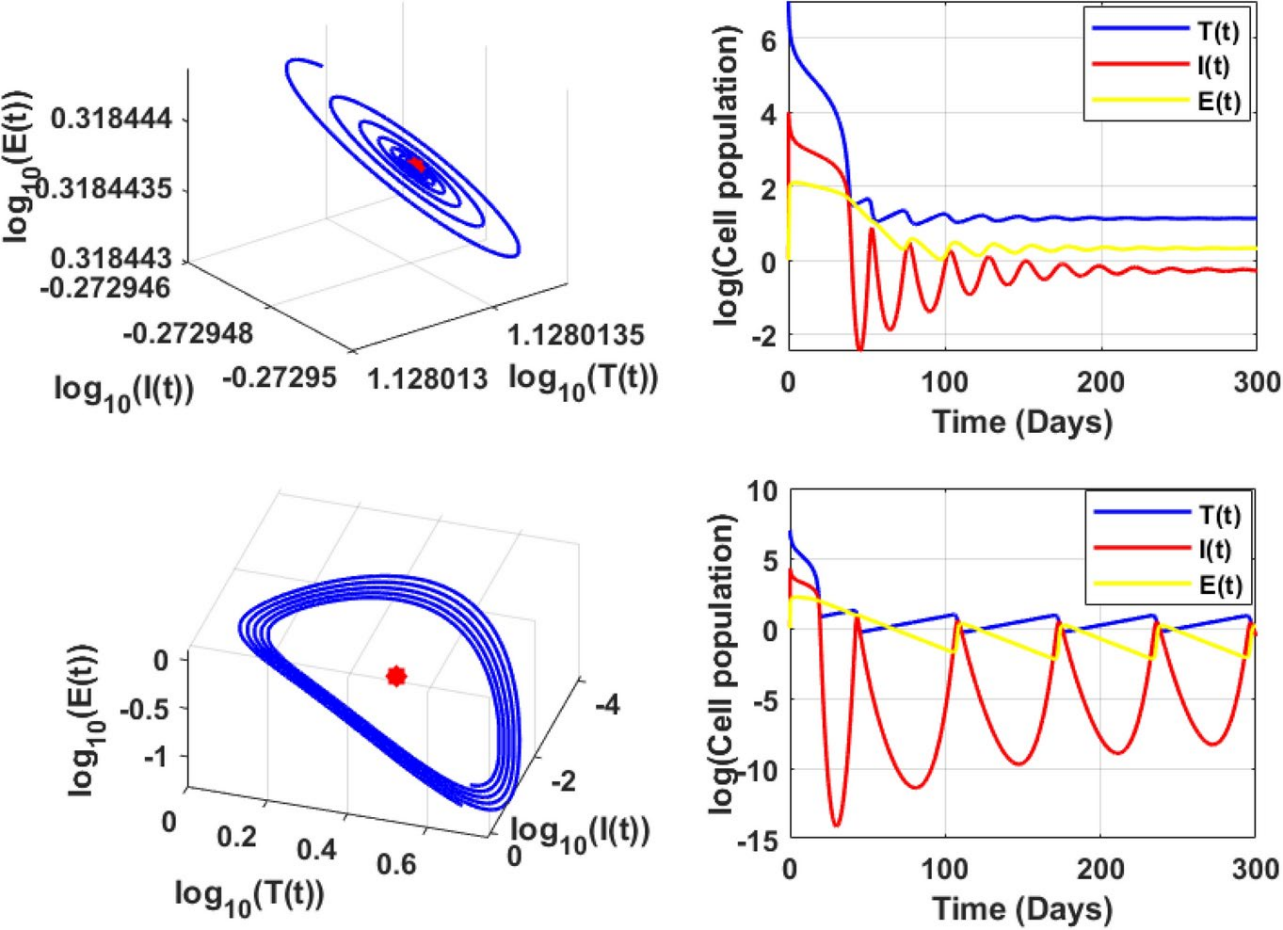
Endemic equilibrium phase portrait and time series for uninfected, infected, and effector cells population, where stable occurs at $\epsilon =0.9$, and oscillation occurs at $\epsilon =0.6$ for the initial condition $T(0)=10^7, I(0)=1, E(0)=1.$.

For $R_0<1,$ there exist atleast one equilibrium point for the parameters, and for $R_0>2$ there exist three equilibrium. Also, for the parameter *c* there exist one or three positive endemic equilibrium. Figure 10 shows the phase portrait and the time series of the endemic equilibrium. Table 6 shows the eigenvalue analysis of endemic equilibrium for the saturated constant *a* and the immune production rate *c*.

**Table 5:** Number of equilibriums with respect to $R_0$, *a* and *c*.

$R_0$ range	$R_0$ value	$a\in (0,0.1)$	$a\in (0.1,0.5)$	$a\in (0.5,2)$	$c\in (0,0.1)$	$c\in (0.1,0.5)$	$c\in (0.5,3)$
		$c=0.1$	$c=0.1$	$c=0.1$	$a=0.1$	$a=0.1$	$a=0.1$
Number of endemic equilibriums							
$R_0<1$	$R_0=0.9$	1	1	1	1	1	3
$R_0=1$	$R_0=1$	1	1	1	1	1	3
$1<R_0<2$	$R_0=1.5$	1	1	1	1	3	3
$R_0>2$	$R_0=2.5$	3	3	3	1	3	3

**Table 6:** Endemic equilibrium: eigenvalue analysis.

Equilibrium	Parameter	Equilibrium	Eigenvalues	Behavior
Endemic	$a = 0.001$	(4.0305; 0.1778; 0.8819)	$(-0.0759,-0.0058 \pm 0.2452i)$	Stable
Endemic	$a = 0.1$	(4; 0.1; 1) (4, 485; 59; 24) (55, 525; 171; 41)	$(-0.0761, -0.0093 \pm 0.2447i )$ $(0.0168, -6.8329, -0.2331)$ $(-0.0258, -10.2283, -0.2396 )$	Bistable
Endemic	$c=0.001$	(1, 474; 57; 2) (69, 234; 344; 6)	$(0.0214,-2.4404, -0.1734)$ $(-0.0366,-3.1402,-0.2134)$	Monostable
Endemic	$c=0.1$	(4; 0.1; 1) (0.4485; 59; 24) (55, 525; 171; 41)	$(-0.0761, -0.0093 \pm 0.2447i)$ $(0.0168,-6.8329,-0.2331)$ $(-0.0258,-10.2283,-0.2396)$	Bistable

**Fig. 11:**
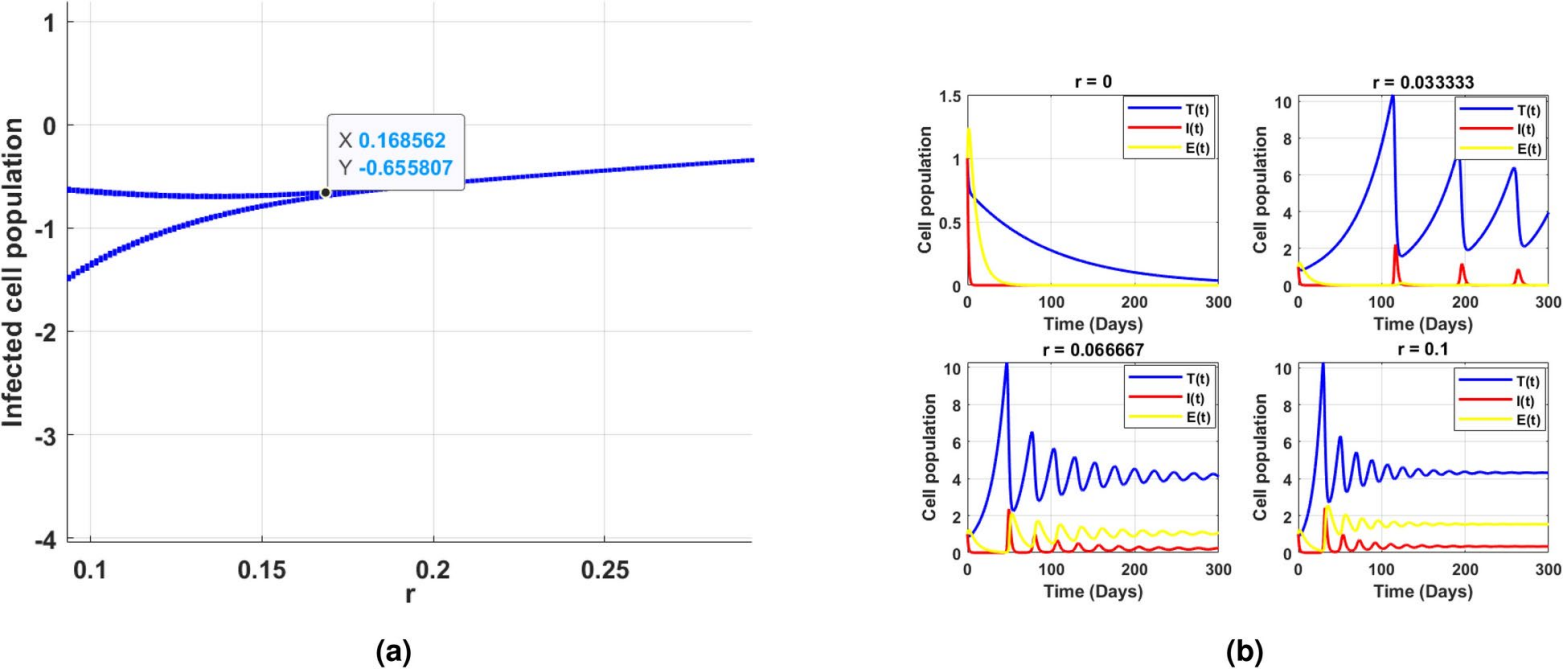
(**a**) shows the existence of Hopf bifurcation behavior in the log scaled infected cell population for increasing and exceeding the threshold of intrinsic growth rate *r*. (**b**) correspondingly illustrates the time series of cell populations for different values of *r*.

**Fig. 12:**
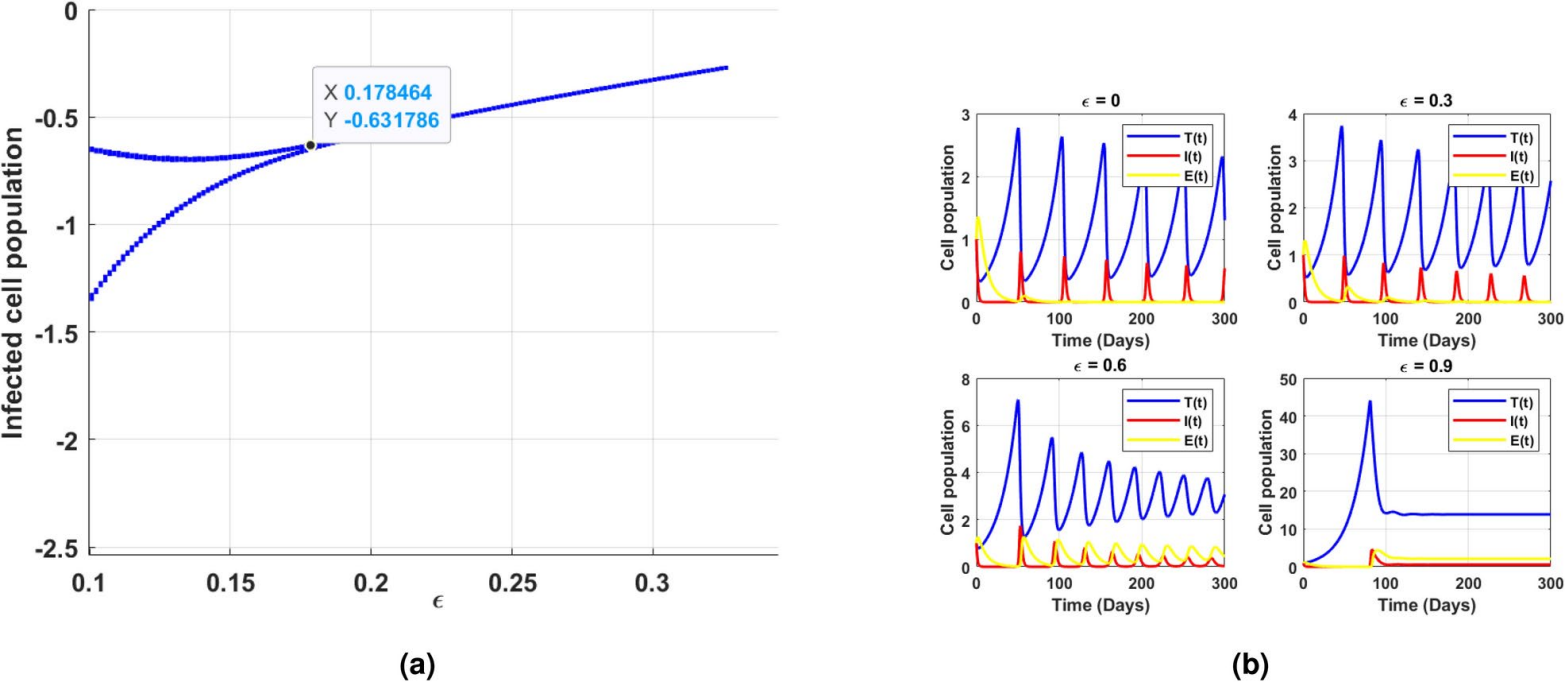
(**a**) Existence of Hopf bifurcation in the log scaled infected cell population *I*(*t*) for the therapy $\epsilon$. (**b**) Time series forecasting of *I*(*t*) with respect to ARTs($\epsilon$). The cell population dynamics can be controlled for the therapy efficacy, if the therapy has low efficacy the cell populations are in oscillations and for higher efficacy of therapy there is a smooth non oscillation behavior in the cell population.

**Fig. 13:**
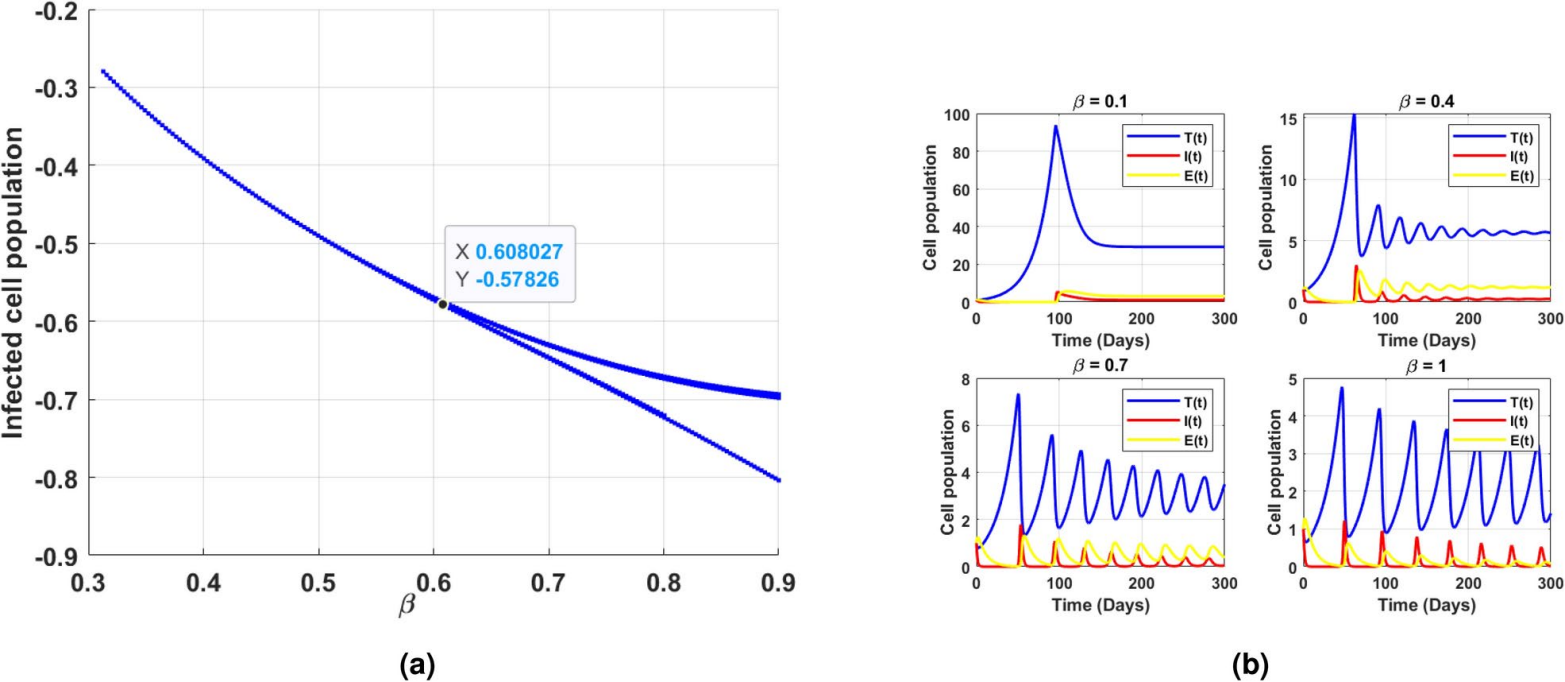
(**a**) Hopf bifurcation in the log scaled infected cell population for the transmission rate $\beta$. (**b**) Time series of the infected cell population for transmission rate $\beta$ for small transmission rate less oscillations, and for the larger transmission rate there is more oscillations in the cell populations.

**Fig. 14:**
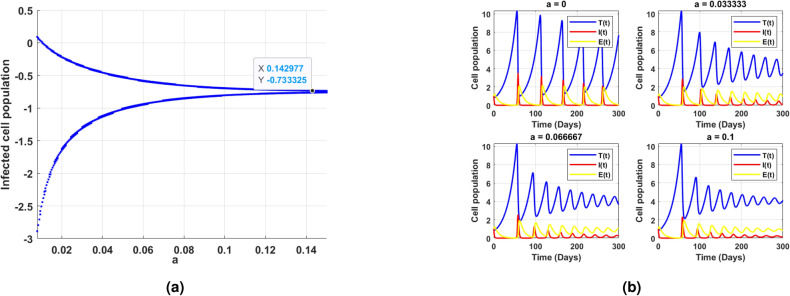
(**a**) Hopf bifurcation in the log scaled infected cell population for the parameter *a*. (**b**) Time series of the infected cell population for different ranges of saturated constant *a*.

**Fig. 15:**
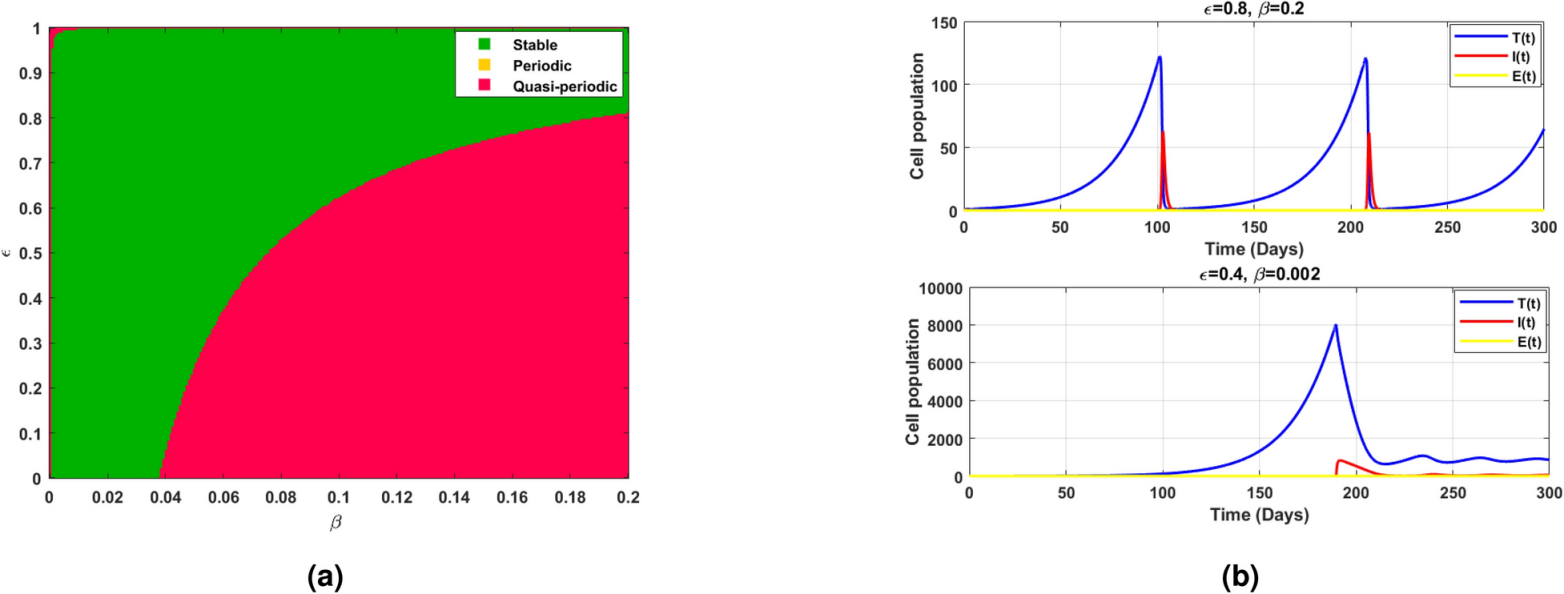
(**a**) The dynamics of the model for the therapy $\epsilon$ and the transmission rate $\beta$, the green region explains the model may exhibits stable behavior but the red region shows quasi-periodic state, that is the behavior cannot be predicted for the larger range. (**b**) Time series for these regions for the different ranges of transmission $\beta$ and therapy $\epsilon$. .

**Fig. 16:**
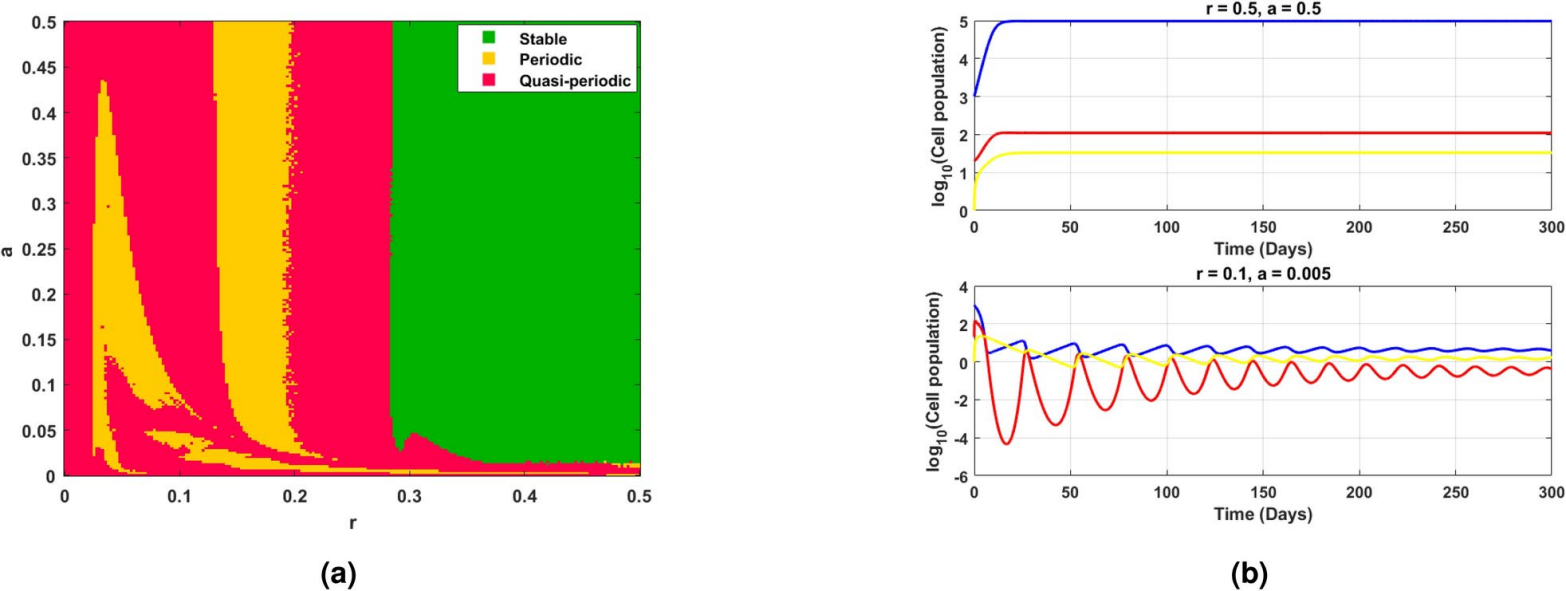
(**a**) The dynamics of the model with intrinsic growth rate *r* and the saturated rate *a*, the green regions shows the model exhibits stable behaviors the red regions illustrates quasi-periodic and the yellow regions stands for periodic behaviors. (**b**) Time series for different ranges of *r* and *a*.

In Figs. 11, 12, 13, and 14 shows the oscillatory behavior in the HIV-1 infection model for the parameters intrinsic growth rate *r*, saturated constant *a*, therapy $\epsilon$ and the transmission $\beta$. Increase in $CD4^+$ population, the efficacy of the therapy, and the inhibition of the transmission at a larger rate, then the oscillations reduced. Similarly, increases in the transmission of large scale show an oscillation in the cell populations. Figure 15a and b, shows two parameters analysis of HIV-1 model with $\epsilon$ and $\beta$, in between values are the state space behaviors of the model (14). Figure 16a, and b shows the two parametric bifurcations for the parameters *r* and *a*. Whereas, the green regions are the stable region and the red regions are the unstable regions. Also, the time series for the stable and periodic regions are shown.

### Backward bifurcation and its types

**Fig. 17:**
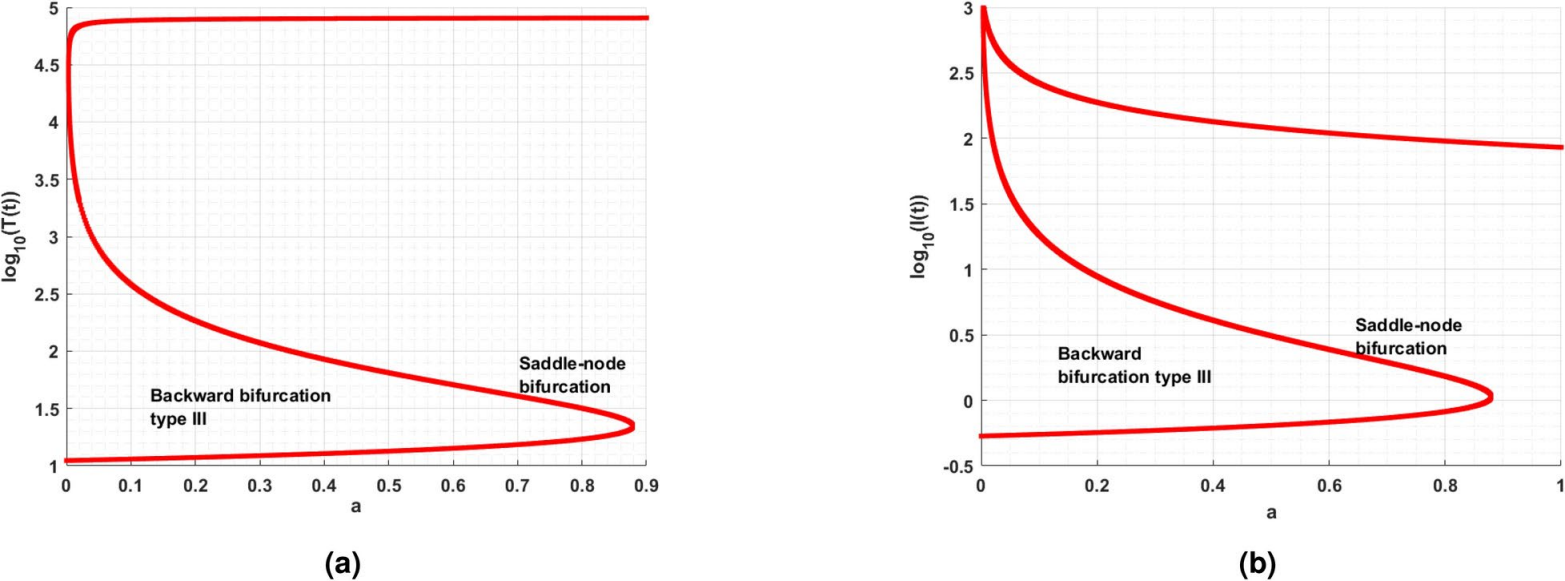
(**a**) The immune-free equilibrium exhibits backward bifurcation type III for the parameter ranges $r = 0.058$, $K = 10^5$, $\beta = 0.9$, $\epsilon = 0.9$, $a \in (0,0.9)$, $c=0.1$. (**b**) Backward bifurcation type III for the infected cell population of immune-free equilibrium.

**Fig. 18:**
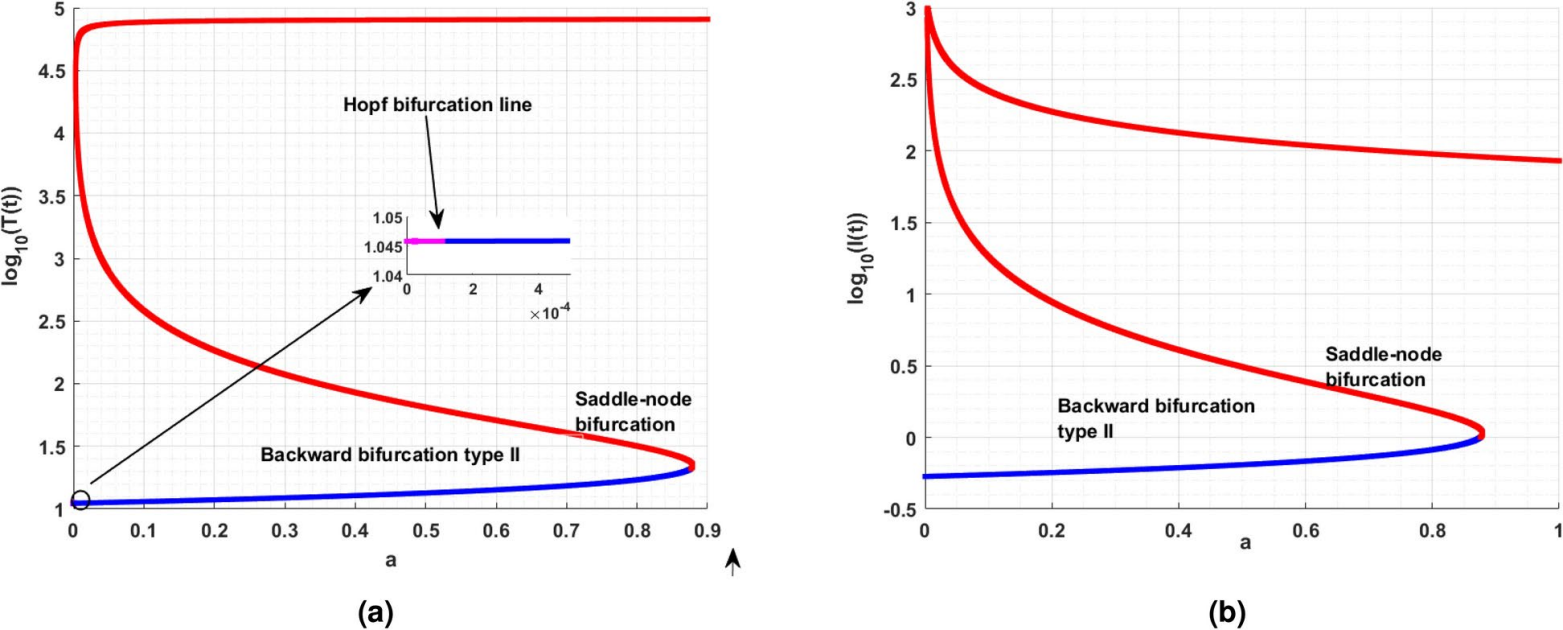
(**a**) and (**b**) Type II backward bifurcation for the immune-free equilibrium $r = 0.058$.

In this section, the backward bifurcation and its types are discussed for the saturated constant *a*. Figure 17a, shows that the immune-free equilibrium especially the target cells and the infected cells undergoes backward bifurcation of type III, that is two unstable equilibria arise from the saddle-node bifurcation and there is an oscillation in the population when the saturated constant *a* nearer to 0.01 and $c=0.1$. This behavior is classified as type III backward bifurcation. Similarly, Fig. 17b shows the type III backward bifurcation for the infected cell population of the immune-free equilibrium. Figure 18a and b shows the backward bifurcation of type II for the uninfected cell population and the infected cell population of the immune-free equilibrium for $c=0.01$. In these figures the red line corresponds to the unstable equilibrium and the blue line is the stable equilibrium. The lower equilibrium is stable and the higher equilibrium is unstable, an intermediate unstable equilibrium separates the both lower and upper equilibrium, this type of behavior is classified as the type II backward bifurcation. The two unstable equilibrium branches from an saddle-node bifurcation, this behaviors is called as backward bifurcation of type II.

### Forward bifurcation for various parameters

**Fig. 19:**
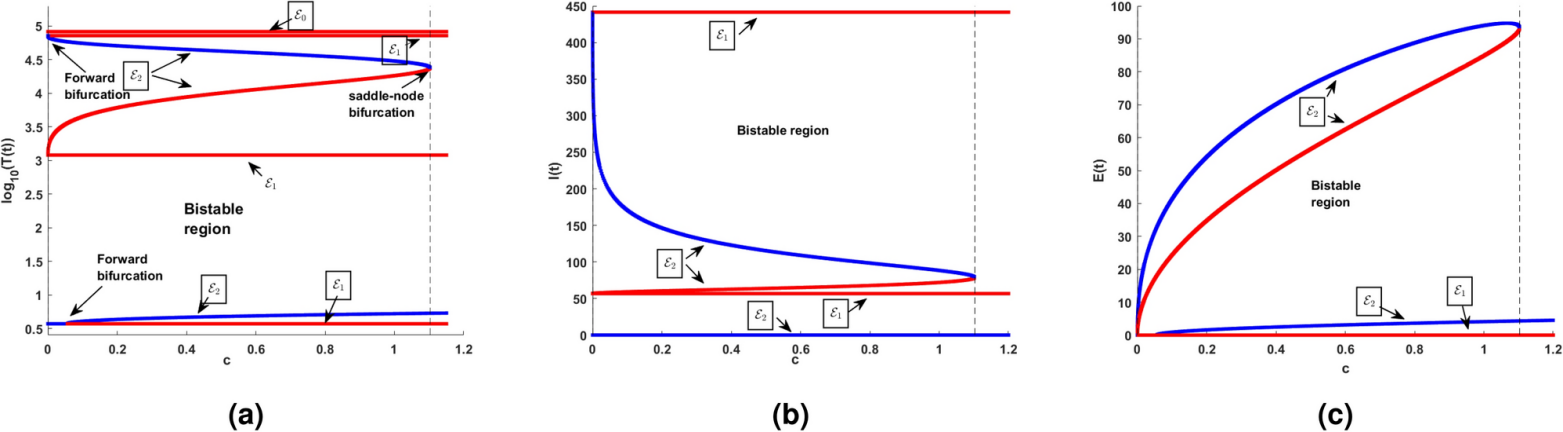
Bistability of both immune-free and endemic equilibrium with respect to *c*.

This section briefly discusses the forward bifurcation of the model (14), with respect to significant parameters immune production rate *c* and saturated constant *a*. The model undergoes the forward bifurcation for the parameter *c* in the target, infection, and effector cell population. Also, the immune-free equilibrium switches its stability from stable to unstable equilibrium point. During the switching of the stability, there is an emergence of the stable endemic equilibrium. Both the lower and the upper endemic equilibrium undergo the forward bifurcation by the emergence of a new endemic equilibrium and there is a switch in the stability. In the higher equilibrium, the emergence of endemic equilibrium collides with an unstable endemic equilibrium destroying and resulting in the saddle-node bifurcation. Also, there is an occurrence of bistability in the endemic equilibrium for more details refer to Fig. 19a–c.

**Fig. 20:**
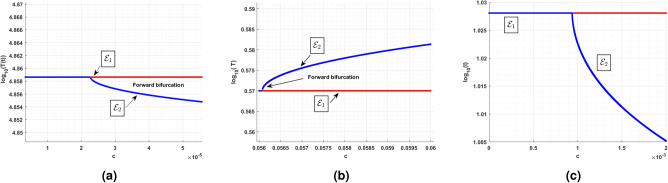
(**a**) Forward bifurcation for the parameters range $r = 0.058, K = 10^5,\beta = 0.9, \epsilon = 0.7,a = 0.1,d_T = 0.01, \delta = 1, m=0.1,\eta = 0.1,$k = 0.1$,b = 0.1$,$c\in (0,0.001).$ (**b**) Forward bifurcation from the lower immune equilibrium and the stable endemic equilibrium arises. (**c**) The forward bifurcation with respect to *c* at $\beta =0.0015$.

**Fig. 21:**
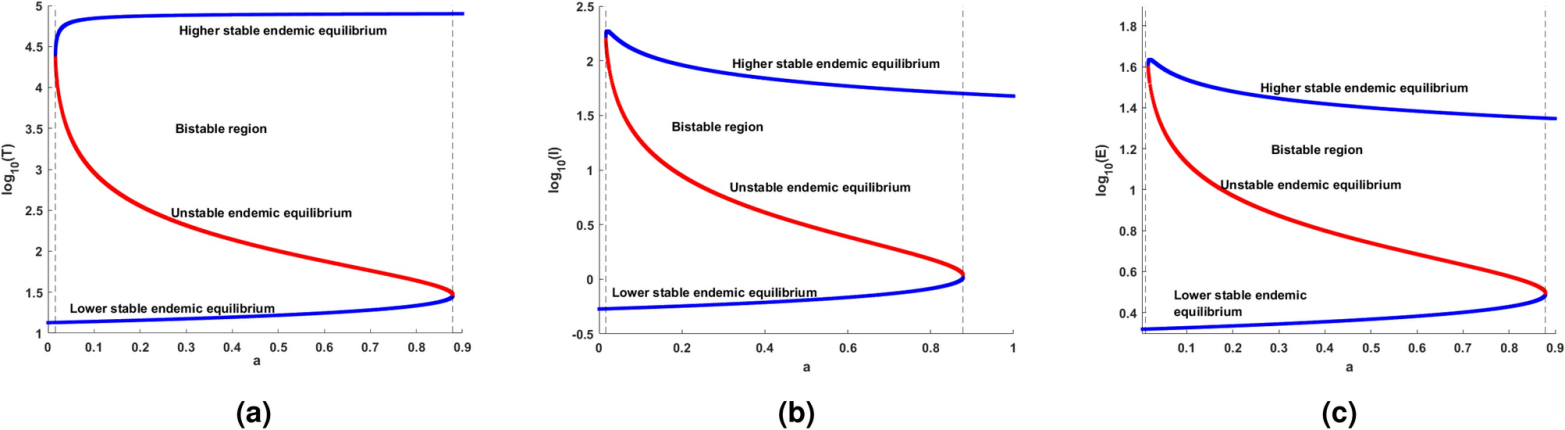
Endemic equilibrium with respect to the parameters $K = 100000$, $\beta = 0.9$, $\epsilon = 0.9$, $a \in (0,0.9)$, $d_T = 0.01$, $\delta = 1$, $m=0.1$, $\eta = 0.1$, $k = 0.1$, $b = 0.1$, $c=0.1$.

Figure 20a–c shows the forward bifurcation of the higher and lower equilibrium, this kind of behavior is called as hysteresis, where the future state depends on the current state or the present state depends on the past. In Fig. 21a–c the bistability of the endemic equilibrium for the target, infected, and effector cell population is studied. In the figures for *a* ranges (0.0001, 0.87), the equilibrium undergoes the bistability in nature. These behaviors are observed in certain patients even after stopping the therapy certain patients can maintain the viral load below the threshold level of less than 50 mL/copies. In the theoretical aspect, this is called a lower equilibrium. There is a slight change in parameter value, system remains in a lower equilibrium. Increasing the parameter value suddenly the system settles in a higher stable equilibrium, which shows an increase in the infected cell population.

**Fig. 22:**
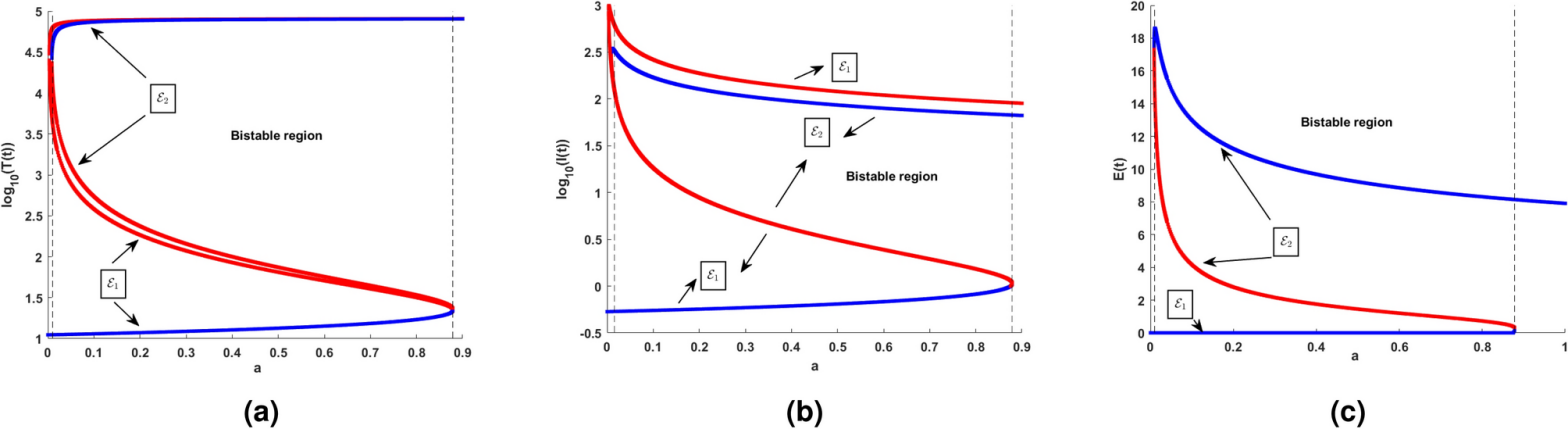
Bistability of both immune-free and endemic equilibrium.

Figure 22a–c show that bistability occurs by both the immune-free equilibrium and the endemic equilibrium. One can see that the system has a lower stable immune-free equilibrium and a higher stable endemic equilibrium, which are separated by the unstable immune and endemic equilibrium.

### Bistability analysis for various parameters

**Fig. 23:**
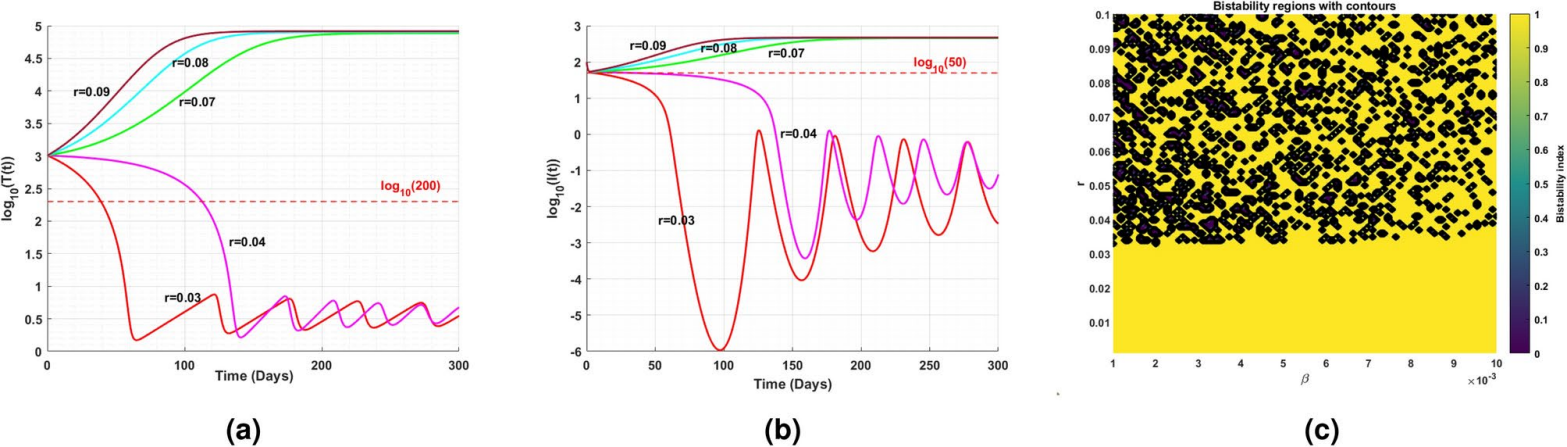
(*a*) The time-series plot shows the bistability of the target cell population for the intrinsic growth rate and the initial conditions $T(0)=1000$, $I(0)=100$, $E(0)=0$. (*b*) The time-series plot shows the bistability of the infected cell population for the intrinsic growth rate and the initial conditions $T(0)=1000$, $I(0)=100$, $E(0)=0$. (*c*) Bistability for the intrinsic growth rate *r* and transmission rate $\beta$. The black colored regions are the bistable regions and the yellow regions include the monostable and oscillatory regions.

Figure 23a, and b shows the bistability of the immune-free equilibrium for the intrinsic growth rate, in the target cell population as the intrinsic growth increases then the target cell population remains in a lower stable equilibrium, then a sudden increase in the population from the lower equilibrium to higher equilibrium. Similarly in the infected cell population, the infected cell exhibits bistability as the target cell population increases there is a possibility of a large number of the cells getting infected resulting in a higher infected equilibrium. Figure 23c shows the bistability regions for the parameters *r* and $\beta$. The black color regions are the bistable region and the remaining regions include the monostable, oscillatory, and unstable regions.

**Fig. 24:**
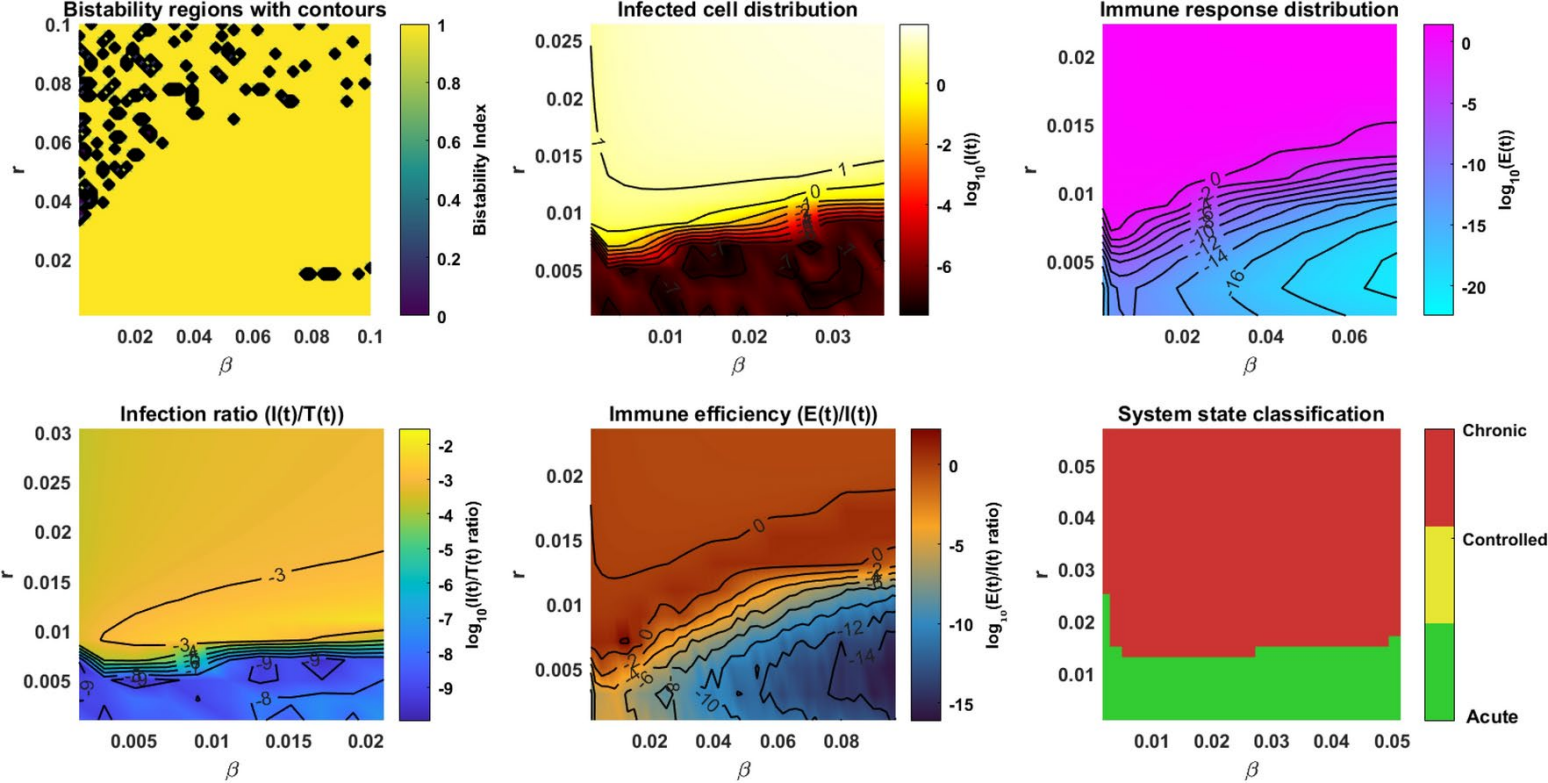
The HIV-1 model distributions and system state analysis.

Figure 24 shows various distributions and system state analysis for the parameters *r* and $\beta .$ In the Fig. 24a the bistability regions are shown, the black regions are the bistable regions and the yellow region is the monostable region. Figure 24b gives the infected cell distribution, the shaded region is suppressed or infected cell populations. Figure 24c is the immune response distribution, the pink region indicates the strong immune response, the effective immune control towards the infection. The purple is the moderate region, where the immune control is partial. The blue region shows the immune exhaustion or immune failure at this stage has the higher risk of AIDS. Figure 24d and e are the infection and immune ratio for the model. In the infection ratio the blue region has lower infection rates, the infection is suppressed and the yellow region has higher transmission of the infection occurs. In the immune ratio, the maroon color region shows higher immune activity, in the dark blue region immune suppression occurs. Figure 24f the green region is the target zone for treatment and the successful ARTs. The red region is the chronic region and higher risk of disease progression.

**Fig. 25:**
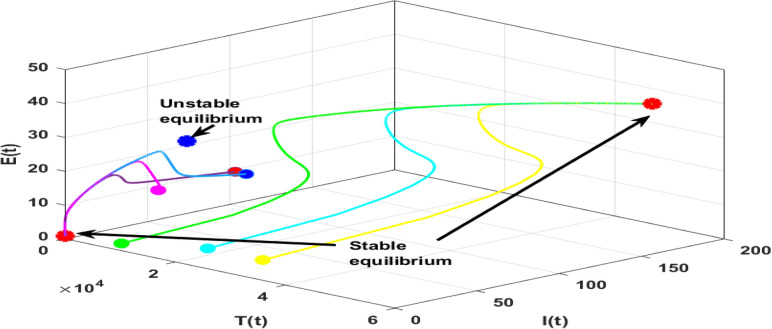
The bistability dynamics of the model for different initial conditions. The red dots are the stable equilibria and the blue dot is the unstable equilibrium. The initial conditions start in pink, maroon, and light blue colors approach the lower stable steady state. The light green color, sky blue color, and yellow color approach the higher stable steady state.

Figure 25, the bistability for the different initial conditions is discussed. The red dot is the stable equilibrium and the blue dot is the unstable equilibrium. For various initial conditions of the cell population, the system states approach the lower and higher stable equilibrium.

There are some limitation in the HIV-1 model. HIV-1 ODE model follows the homogeneity in the population dynamics, ignores the spatial heterogeneity. The model simplify the effect of therapy, the therapy can be time-variant, considering the therapy ranges over time gives the better understanding of the model. Though the HIV-1 model fitted with data there are certain unusual behaviors in the data of certain patients, they were ignored because it is challenging to conclude biologically.

There certain complexities in the structured of the differential model since the model is highly non-linear finding analytical equilibrium is challenging. Hence, this study utilizes the numerical approach to perform steady state analysis. Due to high nonlinearity, finding the solution of the model and perform bifurcation analysis for the HIV-1 model are challenging to analyze. Also, the study performs the data fitting for the data availed from the clinical trials, fitting is time consuming. Existence of multiple states requires careful validation in numerical simulations to analyze the stability, bifurcation and the bistability phenomena in HIV-1 infection.

## Conclusion

The analysis of the HIV-1 infection model, including compartments such as uninfected cells, infected cells, viruses, and effector cell populations, along with dynamics such as growth, transmission, death rates, and immune production rate are performed. The effect of non-monotone incidence rate and the immune response productions has been studied. To ensure the community spread, $R_0$ for the model is derived. The numerical investigation shows the existence of the endemic equilibrium for $R_0<1$ indicates the complex behavior of the HIV-1 model. Hence, the direction of the study focuses on the parameter involving not only $R_0$ but also the immune response production rate *c* and the saturated constant *a* which inhibits the transmission and the therapy. Stability analysis has been focuses concerning three cases such as disease-free, immune-free, and endemic equilibrium utilizing the Routh-Hurwitz criterion. Through sensitivity analysis, the significant parameters that affect the dynamics of HIV-1 infection have been identified. This study workout both theoretical results and the experimental validation in terms of data-driven modeling approach.

Theoretically, qualitative changes such as saddle-node, trans-critical, and Hopf bifurcation conditions have been determined. The study finds the bistability nature in the dynamics of the HIV-1 model for various significant parameters associated with the immune-free and endemic equilibriums. There is a certain behavior in the viral load of patients, for some patients, the viral population remains below the threshold level after the therapy is stopped due to the strong immune responses towards the viruses. This behavior is considered a bistable behavior in the dynamics of HIV-1, the viral population remains in the lower stable steady state for a period. The theoretical results have been validated through numerical investigations to find the equilibrium points, perform the stability analysis, and the qualitative changes in the dynamics of the HIV-1 model. Bistability behavior for the various significant parameters identified using sensitive analysis is discussed in detail such as types of backward bifurcation and forward bifurcation, and the stability switches between the equilibrium for the immune responses, intrinsic growth rates, and saturated constants. The bistability behavior is observed in the dynamics, and the biological aspects of the bistability behavior with the validation of the data are investigated. Moreover, the bistability of the system trajectories for various stages of infection has been numerically explored.

In the near future, the model can be extended by considering more biological aspects such as latency in the infection, drug resistance towards the therapy, and immune impairment. The clinical data often contains noises hence the study can be extended to a stochastic model. The infected cell dynamics in HIV-1 is a long process, hence it takes years to achieve the AIDS stage almost 8-10 years, the infected cell population varies also concerning age and time, therefore the HIV-1 model can be extended to an age-structured model.

## Data Availability

All data generated or analysed during this study are included in this published article.
